# Facile synthesis of recyclable polythioimidocarbonates *via* aromatization-driven alternating copolymerization of *para*-quinone methide and isothiocyanates†

**DOI:** 10.1039/d5sc00050e

**Published:** 2025-02-21

**Authors:** Wen-Dao Chu, Si-Yu Dan, Jie Zhan, Bo Chen, Ji Xian, Chun-Mei Wang, Quan-Zhong Liu, Jincai Wu, Chun-An Fan

**Affiliations:** a Precise Synthesis and Function Development Key Laboratory of Sichuan Province, College of Chemistry and Chemical Engineering, China West Normal University No. 1 Shida Road Nanchong Sichuan 637002 China chuwendaonpo@126.com; b State Key Laboratory of Natural Product Chemistry, College of Chemistry and Chemical Engineering, Lanzhou University 222 Tianshui Nanlu Lanzhou 730000 China wujc@lzu.edu.cn fanchunan@lzu.edu.cn

## Abstract

The efficient and controllable alternating copolymerization of *para*-Quinone Methide (*p*-QM) is rare and challenging. The aromatization-driven alternating copolymerization of *p*-QM with isothiocyanates is explored for the first time under mild conditions. In the presence of the key catalyst *m*-phthalic acid and the initiator TBD, the reaction can efficiently produce completely alternating polythioimidocarbonates with narrow molecular weight distributions and high molar mass (up to 103.6 kg mol^−1^). Experimental studies and DFT calculations suggest that *m*-phthalic acid plays a synergistic catalytic role. Remarkably, copolymers can be recycled back into monomers with excellent yields under vacuum at a temperature of 190 °C in just a few minutes without solvents or catalysts.

## Abstract 2

For the first time, the aromatization-driven alternating copolymerization of *p*-QM with isothiocyanates is explored under mild conditions.

## Graphical abstract

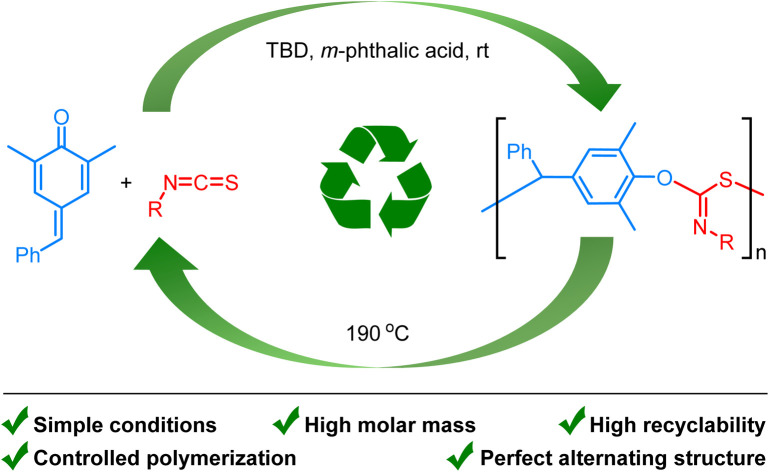

Owing to the inexpensive availability, processing versatility and exceptional mechanical properties, synthetic polymers have found numerous applications across various domains of our daily lives. The extensive production and unregulated disposal of plastics, however, have led to significant economic and environmental repercussions due to their limited degradation and recyclability.^1–6^ Efforts to tackle these challenges include mechanical recycling,^7–9^ upcycling towards value-added chemicals,^10–14^ and chemical recycling to monomers.^15–18^ Among these approaches, chemical recycling to monomers is particularly appealing due to its potential for regenerating polymers with virgin-quality and reducing the demand for raw materials.^19–31^ To address the end-of-life challenge of polymers and safeguard finite natural resources, the development of a circular plastics economy through chemical recycling to monomers is essential for advancing sustainability efforts.^32,33^

Sulfur-containing polymers exhibit exceptional characteristics, including optical, mechanical, and metal-adsorbing properties.^34,35^ Consequently, significant efforts have been dedicated to the preparation of such polymers.^36–49^ Among these methods, the ring-opening copolymerization (ROCOP) of strained heterocycles with isothiocyanates stands out as one of the most efficient approaches (Scheme 1a).^50–56^ The Feng, Xiong, Wu, and Zhao groups independently reported the synthesis of polythioimidocarbonates through the ROCOP of isothiocyanates and epoxides.^50–52^ Subsequently, the scope of heterocycle monomers was expanded to episulfides and aziridines by other groups.^53–55^ Very recently, Plajer and coworkers reported the alternating copolymerization of oxetanes and isothiocyanates in the presence of a heterobimetallic catalyst.^56^ Despite these elegant achievements, the recycling of the polymers was not investigated. In addition, all the reactions are driven by the relief of ring strain. Given the numerous applications of sulfur-containing polymers and the escalating issue of plastic pollution, there is an urgent need to explore new monomers and concepts for synthesizing recyclable sulfur-containing polymers.

**Scheme 1:**
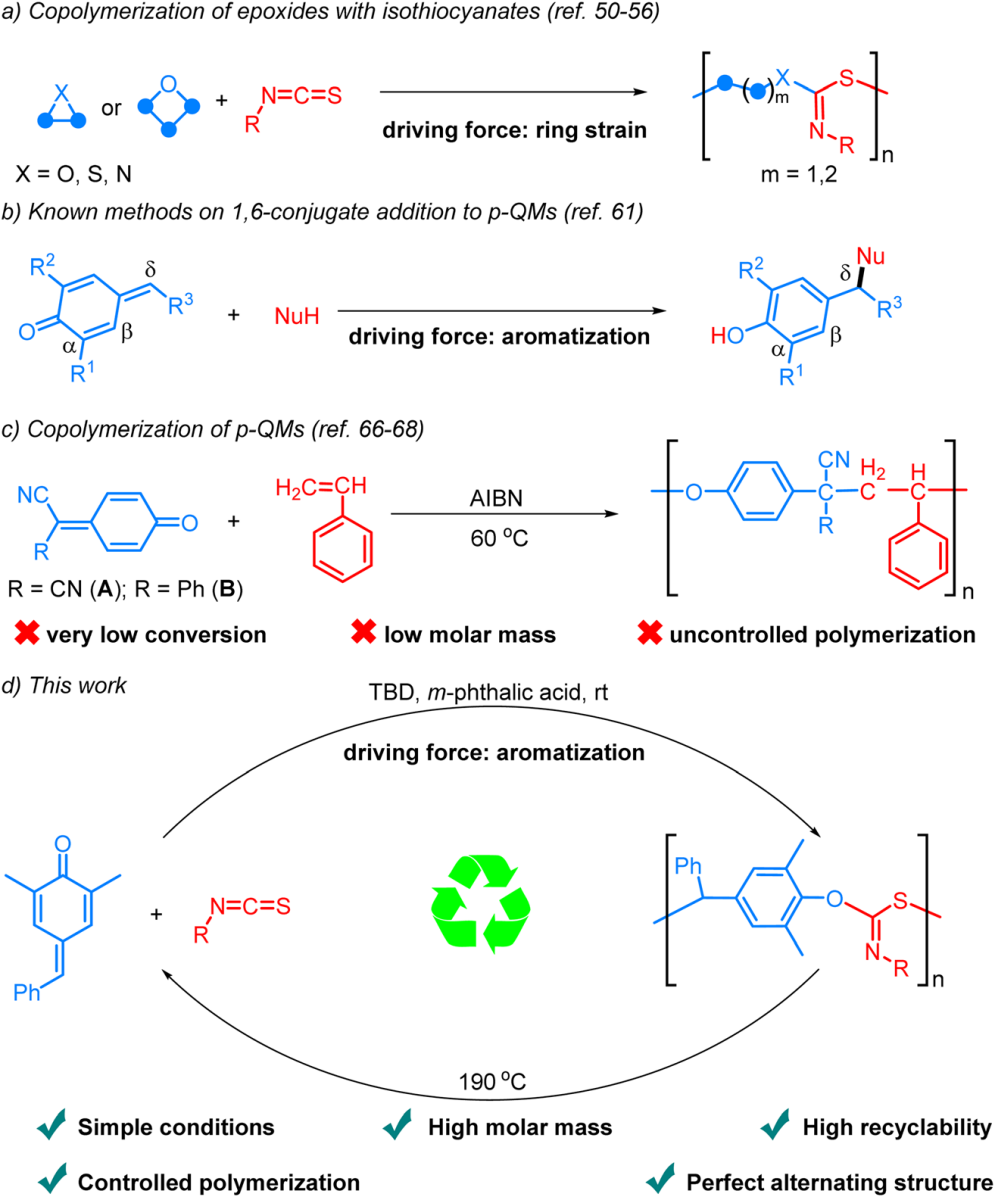
Previous studies and our reaction design.

*para*-Quinone methides (*p*-QMs), which are structurally characterized by the unique assembly of carbonyl and olefinic moieties, have been widely observed in various natural products and recognized as reactive intermediates in organic synthesis, materials chemistry and biological processes.^57,58^ The canonical cyclohexadienone form exhibits a pronounced inclination towards aromatization, thereby imparting enhanced electrophilicity and facilitating 1,6-conjugate addition.^59^ Significant progress has been achieved in the past decade regarding the development of highly efficient catalytic systems that facilitate the addition of diverse nucleophiles to the δ position of *p*-QMs through an aromatization driving force (Scheme 1b).^60,61^ Significantly, the homopolymerization of *p*-QMs *via* 1,6-conjugate addition has also been disclosed.^62–65^ However, there are very few reports on the alternating copolymerization of *p*-QMs due to their exceptionally high reactivity and tendency for homopolymerization, resulting in the formation of copolymers in a random fashion. By taking advantage of the fact that *p*-QMs (A, B) are not homopolymerizable with radical initiators, Itoh and coworkers reported the interesting radical alternating copolymerization of *p*-QMs (A, B) with styrene, but the process exhibited limited conversion rates, low molar mass, and uncontrolled polymerization (Scheme 1c).^66,67^ In the case of 7-cyano-7-(ethoxy carbonyl)-1,4-benzoquinone methide, which was capable of undergoing homopolymerization in the presence of a free radical initiator, it could only copolymerize with styrene in a random fashion.^68^ Therefore, the development of efficient and controllable alternating copolymerization of *p*-QMs remains a great challenge. To achieve such reactions, two challenges must be overcome: firstly, the identification of suitable co-monomers exhibiting higher activity than *p*-QMs to inhibit their homopolymerization; secondly, the exploration of an appropriate catalytic system capable of activating the monomers, enhancing reaction efficiency, and simultaneously stabilizing the chain ends to prevent undesired side reactions. Considering the high reactivity and limited tendency for homopolymerization of isocyanates, we envisioned that they might be ideal co-monomers for achieving alternating copolymerization with *p*-QMs. Herein, we report the first example of aromatization-driven anionic alternating copolymerization of *p-*QM and isothiocyanates under mild conditions. In the presence of the key catalyst *m*-phthalic acid and the initiator TBD, the reaction can efficiently produce completely alternating polythioimidocarbonates with narrow molecular weight distributions and high molar mass (up to 103.6 kg mol^−1^, Scheme 1d). By utilizing the reversibility of 1,6-conjugate addition reactions of *p*-QMs,^69–72^ the copolymers can be recycled back into monomers with excellent yields under vacuum at 190 °C in just a few minutes without solvents or catalysts.

The organocatalytic alternating copolymerization of *p*-QM and isothiocyanate 1a was initially investigated with commonly used organic bases, namely 4-dimethylaminopyridine (DMAP), 1,8-diazabicyclo[5.4.0]undec-7-ene (DBU), and 1,5,7-triazabicyclo[4.4.0]dec-5-ene (TBD) (Table 1). To our delight, this alternating copolymerization proceeded smoothly and achieved more than 90% conversion in 1 h with a [*p*-QM] : [1a] : [base] ratio of 100 : 400 : 1 in 0.5 M THF at room temperature (for screening of the ratio of *p*-QM to isothiocyanate, see the ESI†). The obtained polymers, however, exhibited slightly broad molecular weight distributions (*Đ*) and number-average molecular weight (*M*_*n*,GPC_) ranging from 60.7–72.5 kDa, which were considerably greater than the calculated theoretical *M*_*n*,theo_ (Table 1, entries 1–3). The results indicated that the initiation efficiency was relatively low when an organobase was employed as an initiator alone. Upon addition of 4-methoxyphenol or benzyl alcohol as chain transfer agents *via* the proton transfer reactions between the phenoxide intermediates and 4-methoxyphenol/benzyl alcohol,^73–75^ the *M*_*n*_ of the polymer significantly decreased but molecular weight distributions of the resulting polymers were still high (entries 4 and 5). This may be caused by a low initiation rate of organobase and side transesterification reactions. To let the molecular weight of the resulting polymer be under control further, it is necessary to relatively increase the main alternating polymerization rate and thereby suppress the side reactions. Thus, the incorporation of acid catalysts may be a potentially viable strategy: on the one hand, the utilization of an acid catalyst can activate *p*-QM, thereby enhancing the initiation efficiency; on the other hand, an acid catalyst can protonate a phenoxide intermediate into less nucleophilic phenol, and then undesired transesterification type side reactions become more difficult. However, the use of *p*-methoxybenzoic acid 2a, *p*-phthalic acid 2b, or *o*-phthalic acid 2c as catalysts did not significantly improve the polymerization reaction (*Đ* = 1.40–1.47, entries 6–8). Gratifyingly, *m*-phthalic acid 2d provided the lowest dispersity (*Đ* = 1.23) and exhibited good agreement between experimental and theoretical *M*_*n*_ (entry 9). In order to gain insights into the nature of *m*-phthalic acid 2d in the copolymerization reaction, we employed ^1^H NMR spectroscopy to observe its interaction with *p*-QM. The protons of *p*-QM exhibited a downfield shift upon treatment with 0.1 equiv. of 2d, indicating that the *p*-QM monomer was activated by 2d for the copolymerization reaction (Fig. S12†). Subsequently, various carboxylic acids were investigated for the copolymerization of *p*-QM and isothiocyanate 1a at a fixed [*p*-QM]_0_/[1a]_0_/[TBD]_0_/[acid]_0_ ratio of 100 : 400 : 1 : 1 (Table S9†). The results showed that both strong and weak carboxylic acids exhibited larger *Đ*s compared to *m*-phthalic acid 2d. The good controlling behavior of 2d may be attributed to its synergistic effect rather than its unique acidity.

**Table 1:** Alternating copolymerization results of *para*-quinone methide and isothiocyanates^a^

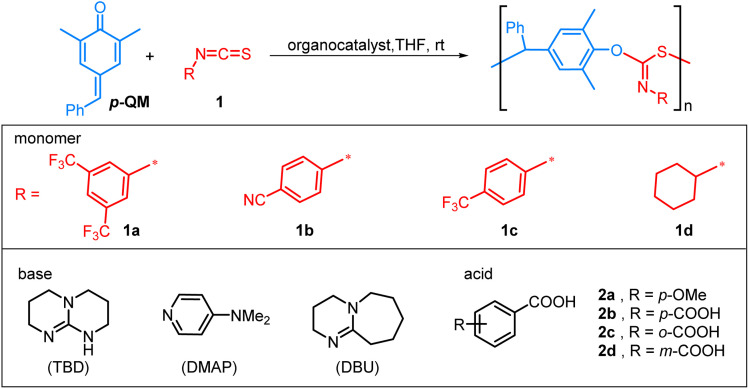						
Entry	Feeding	Time (h)	Conv.^b^ (%)	*M*_*n*,theo_ c (kDa)	*M*_*n*,GPC_ d (kDa)	*Đ* d
1	*p*-QM/1a/DMAP (100/400/1)	1	93	44.9	68.9	1.57
2	*p*-QM/1a/DBU (100/400/1)	1	91	43.9	72.5	1.59
3	*p*-QM/1a/TBD (100/400/1)	1	99	47.8	60.7	1.47
4	*p*-QM/1a/TBD/4-methoxyphenol (100/400/1/1)	0.6	99	47.8	27.1	1.46
5	*p*-QM/1a/TBD/benzyl alcohol (100/400/1/1)	5	93	44.9	45.7	1.43
6	*p*-QM/1a/TBD/2a (100/400/1/1)	1	91	43.9	54.7	1.46
7	*p*-QM/1a/TBD/2b (100/400/1/0.5)	1	91	43.9	50.9	1.47
8	*p*-QM/1a/TBD/2c (100/400/1/0.5)	1	91	43.9	40.3	1.40
9	*p*-QM/1a/TBD/2d (100/400/1/0.5)	0.8	97	46.8	51.2	1.23
10	*p*-QM/1a/TBD/2d (25/100/1/0.5)	0.2	96	11.7	13.6	1.15
11	*p*-QM/1a/TBD/2d (50/200/1/0.5)	0.3	99	23.9	29.6	1.16
12	*p*-QM/1a/TBD/2d (200/800/1/0.5)	6	96	92.5	103.6	1.25
13	*p*-QM/1b/TBD/2d (100/400/1/0.5)	2	99	36.8	28.0	1.40
14	*p*-QM/1c/TBD/2d (100/400/1/0.5)	1.5	99	41.1	29.5	1.36
15	*p*-QM/1d/TBD/2d (100/400/1/0.5)	2	89			

a The copolymerization was conducted in THF in a glovebox at 25 °C, [*p*-QM]_0_ = 0.5 M.

b Conversion of *p*-QM, determined by ^1^H NMR spectroscopy.

c Calculated molar mass based on thw [*p*-QM]_0_/[I]_0_ ratio and conversion.

d *M*_*n*_ and *Đ* were determined by GPC analysis in THF.

The controlled nature of copolymerization was validated by conducting the reaction using varying ratios of [*p*-QM]_0_/[TBD]_0_ ranging from 25 to 200. The polymerizations all achieved high conversions. *M*_*n*,GPC_ of the polymers increased linearly with increasing [*p*-QM]_0_/[TBD]_0_ ratio, while maintaining low *Đ*s (1.15–1.25) (entries 9–12, Fig. 1a and b). Notably, at [*p*-QM]_0_/[TBD]_0_ = 200 : 1, the resulting polymer can achieve a number average molecular weight of 103.6 kDa with a dispersity of 1.25 (entry 12). The alternating sequence of the resulting *p*-QM/1a copolymer was confirmed through MALDI-TOF MS. As shown in Fig. 1c, four distributions of equal intervals were observed, and the interval value (*m*/*z* = 481.4) matches the mass of *p*-QM and 1a repeating units. Therefore, it can be inferred that a polymer chain exhibits a highly alternating sequence of *p*-QM and 1a. The four populations correspond to two different chain ends, with two of them having an extra *p*-QM unit. The two populations terminate with 1a, which can be described by using the formula [TBD + (*p*-QM + 1a)_*n*_] associated with two distinct cations of H^+^ or NH_4_^+^. The other two populations end with *p*-QM, characterized by the equation [TBD + (*p*-QM + 1a)_*n*_ + *p*-QM], and are also linked to either H^+^ or NH_4_^+^. The presence of a TBD molecule in the polymer chains indicates that copolymerization is initiated by TBD, which can be further confirmed by the distinct characteristic peaks of TBD in the ^1^H NMR spectrum of the resulting polymer when the ratio of the monomer to TBD is 15 (Fig. S10†). The structure of the copolymer was further analyzed by ^1^H NMR, ^13^C NMR and FTIR. As shown in Fig. 2a, the molar ratio of *p*-QM to 1a in the copolymer is 1 : 1 and the characteristic resonances of *p*-QM homopolymerization at 5.6 ppm (Fig. S11†) were not observed, indicating a completely alternating structure. The presence of a ^13^C NMR signal at 150 ppm (Fig. 2b) confirmed the formation of O(S)C=N units instead of O(N)C=S, which exhibited a signal at around 187 ppm.^76^ Additionally, FTIR analysis further supported the existence of O(S)C=N moieties. A prominent peak at 1630 cm^−1^ corresponding to the C=N group was observed, while no characteristic IR absorption of the C=S group at approximately 1500 cm^−1^ was detected (Fig. S3†).^76^ Besides 1a, this method can be readily adapted for the copolymerization of other aromatic isothiocyanates with good control, including 4-CNC_6_H_4_ substituted isothiocyanate 1b and 4-CF_3_C_6_H_4_ substituted isothiocyanate 1c (entries 13 and 14). The resulting polythioimidocarbonates also demonstrate a perfectly alternating structure (Fig. S4–S9†). However, the copolymerization of *p*-QM with aliphatic isothiocyanate 1d was unsuccessful, only yielding the *p*-QM homopolymer (entry 15, Fig. S11†). This can be attributed to the aromatic group's stronger electron-withdrawing ability compared to that of the alkyl group, thereby resulting in higher reactivity of aromatic isothiocyanates compared to aliphatic counterparts.

**Fig. 1:**
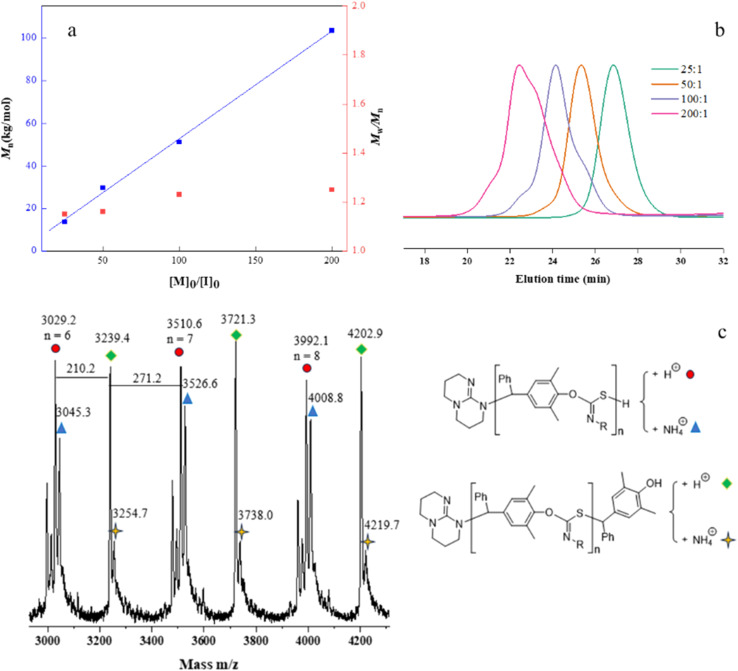
(a) Plots of *M*_*n*_ and *Đ vs.* the [*p*-QM]_0_/[TBD]_0_ ratio; (b) SEC traces of copolymers (color online); (c) MALDI-TOF MS spectrum of the low-molecular-weight P(*p*-QM-alt-1a).

**Fig. 2:**
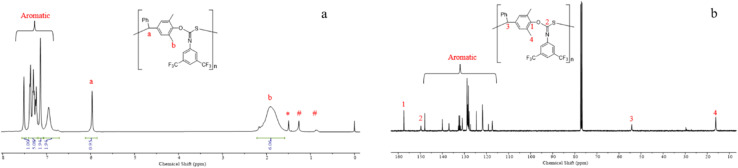
(a) ^1^H NMR (CDCl_3_) spectrum of P(*p*-QM-alt-1a) obtained by using [*p*-QM]/[1a]/[TBD]/[2d] = 100/400/1/0.5; (b) ^13^C NMR (CDCl_3_) spectrum of P(*p*-QM-alt-1a) obtained by using [*p*-QM]/[1a]/[TBD]/[2d] = 100/400/1/0.5 (*H_2_O, ^#^*n*-hexane).

To gather in-depth insight into the catalytic mechanism, we carried out several kinetic experiments and measured the monomer conversion *via*^1^H NMR. As shown in Fig. 3a, the semilogarithmic plot displayed a linear relationship (*R*^2^ = 0.99) indicative of a first order in *p*-QM concentration. By varying the initial concentration of isothiocyanate 1a from 1.5 to 3.0 M without altering other reaction conditions, a zero-order dependence on 1a concentration was confirmed (Fig. 3b). This suggests that 1a insertion is not the rate-determining step during the copolymerization process. Next, the initial TBD concentration was varied to explore the correlation between *k*_obs_ and [TBD]_0_, and all copolymerizations exhibited distinct first-order kinetics (Fig. S7†). The plot of ln(*k*_obs_) against ln[TBD]_0_ was linear with a gradient of 1.2 (Fig. 3c). It is noteworthy that an increase in the initial concentration of *m*-phthalic acid 2d resulted in a decrease in the reaction rate. A double logarithmic plot of the *k*_obs_ as a function of [2d]_0_ was fit to a straight line with a slope of −0.2 (Fig. 3d). We reasoned that the formation of an acid–base complex between 2d and TBD could potentially reduce the reaction rate induced by TBD.^77^

**Fig. 3:**
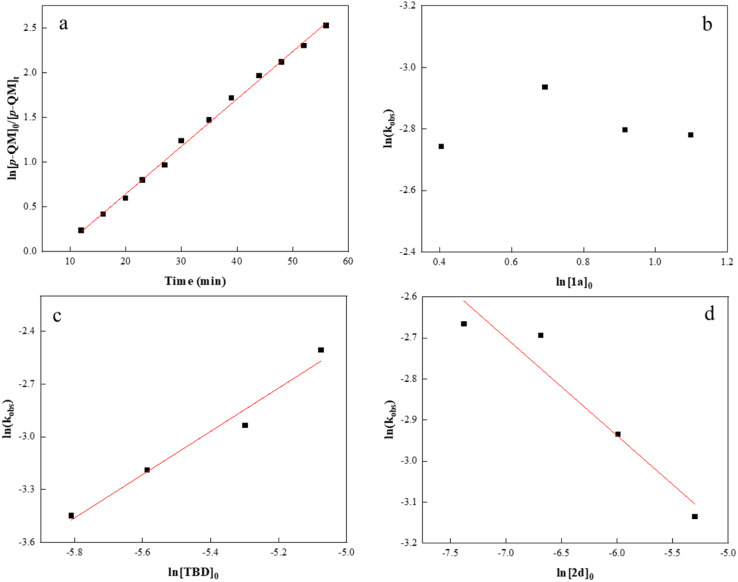
Kinetics for *p*-QM/isothiocyanate 1a copolymerization: (a) kinetic plot of ln([*p*-QM]_0_/[*p*-QM]_*t*_) *versus* time at a [*p*-QM]_0_/[1a]_0_/[TBD]_0_/[2d]_0_ ratio of 100 : 400 : 1 : 0.5. (b) Plot of *k*_obs_*versus* [1a]_0_. (c) Plot of ln(*k*_obs_) *versus* ln[TBD]_0_. (d) Plot of ln(*k*_obs_) *versus* ln[2d]_0_.

Based on the experimental results obtained above, a plausible alternating copolymerization mechanism is proposed in Scheme 2. First, *p*-QM interacts with *m*-phthalic acid through hydrogen-bonding, followed by the nucleophilic attack of TBD to generate a phenol intermediate (I) driven by aromatization energy. The side reactions of transesterification and homopolymerization of *p*-QM are effectively suppressed due to the relatively weaker nucleophilicity of phenol compared to phenoxide. Subsequently, I reacts easily with isothiocyanates on the electron-deficient carbon to produce a sulfur anion II (–C(=NR)S–). After the formation of a hydrogen bond between a fresh *p*-QM and another carboxyl group of *m*-phthalic acid, the activated *p*-QM is subjected to attack by the sulfur anion II. Thereafter, isothiocayanates and *p*-QM are alternatingly inserted to carry out the copolymerization, ultimately affording polythioimidocarbonates.

**Scheme 2:**
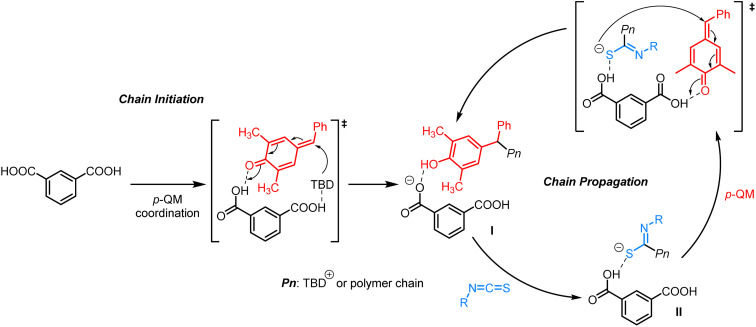
Proposed mechanism for alternating copolymerization of *p*-QM and isothiocyanates.

DFT calculations were also conducted to gain a better mechanistic insight into the proposed alternating copolymerization mechanism (Scheme 3).^78,79^ In the initial period, the *p*-QM monomer forms a hydrogen bond with *m*-phthalic acid (INT1), resulting in a slight increase in Gibbs free energy compared with the initial state. After overcoming an activation free energy barrier of 14.56 kcal mol^−1^ (TS1), a phenol intermediate INT2 is obtained through the nucleophilic addition of TBD to the activated *p*-QM with a relative energy of −2.99 kcal mol^−1^. To enhance computational efficiency, the addition product of TBD and *p*-QM was approximated as 2,4,6-trimethylphenol. The insertion of isothiocyanate 1a and the intermediate INT2 spontaneously transforms into a more stable intermediate INT3, in which the phenol forms two hydrogen bonds with *m*-phthalic acid. A sulfur anion INT4 is formed following the nucleophilic addition of 2,4,6-trimethylphenol to the electron-deficient carbon of isothiocyanate through TS2 with an energy barrier of 11.97 kcal mol^−1^. After a hydrogen bond is formed between an additional *p*-QM and *m*-phthalic acid (IN5 to IN6), the nucleophilic addition of a sulfur anion to the activated *p*-QM affords INT8 by overcoming an activation free energy barrier of 19.06 kcal mol^−1^ (TS3 to INT8). Thus, the two carboxyl groups in *m*-phthalic acid play a synergistic catalytic role; one activates *p*-QM while the other stabilizes the nucleophilic phenoxide or sulfur anion during the propagation process. The energy profiles reveal that the nucleophilic addition of the sulfur anion to *p*-QM presents a significantly high total energy barrier of 19.06 kcal mol^−1^, thereby identifying this step as the rate-determining step. This conclusion is in agreement with the kinetic study results presented in Fig. 3.

**Scheme 3:**
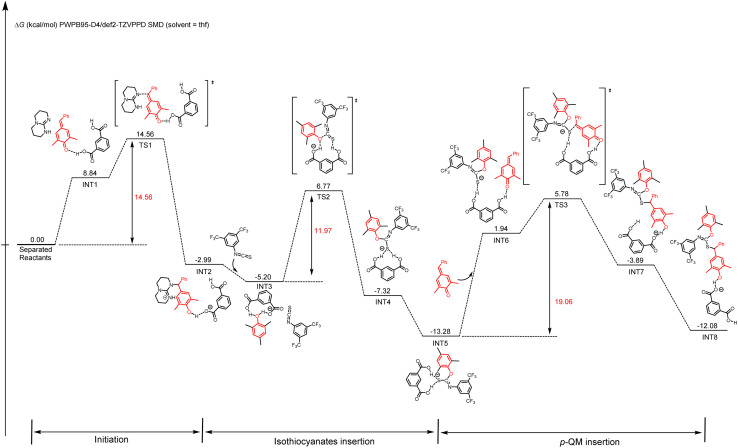
Energy diagram of *p*-QM and 1a copolymerization initiated by TBD.

To assess the recyclability of polythioimidocarbonates, we next examined the depolymerization capability using a copolymer comprising *p*-QM and 1a as an illustrative example. This polymer was successfully depolymerized back to *p*-QM and 1a with 92% yield using a commercially available sublimation device under vacuum at a temperature of 190 °C in just 2 minutes, without the need for solvents or catalysts (Fig. 4). Thermogravimetric analysis (TGA) of these polymers showed that the thermal decomposition of all the copolymers initiates at approximately 175 °C (Fig. S13B–S15B†), which agrees with the recyclable behaviors at about 190 °C. The glass transition temperatures (*T*_g_s) of these copolymers were surprisingly not observed on the differential scanning calorimetry (DSC) curves (Fig. S13A–S15A†). We speculated that the *T*_g_s might be higher than their decomposition temperature because of their hard and brittle properties.

**Fig. 4:**
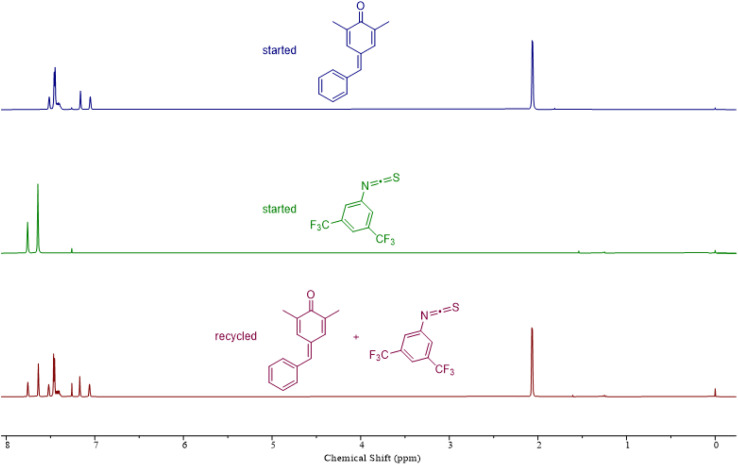
Recyclability study of the copolymer consisting of *p*-QM and 1a.

In conclusion, we disclose the first example of alternating copolymerization of *p*-QM and isothiocyanates driven by aromatization under mild conditions. This method provides completely alternating polythioimidocarbonates with narrow molecular weight distributions and high molar mass. The utilization of *m*-phthalic acid as a catalyst is crucial for the reaction. Experimental studies and DFT calculations suggest that *m*-phthalic acid plays a synergistic catalytic role. Remarkably, the copolymers can be completely recycled back into monomers with excellent yields under vacuum at a temperature of 190 °C in just a few minutes without solvents or catalysts. Further studies are currently underway in our laboratory to improve the physical properties of these copolymers and develop copolymerization of *p*-QM with other monomers.

## Data availability

The data supporting this article have been included as part of the ESI.†

## Author contributions

C. W.-D. and D. S.-Y. performed and analyzed the experiments. Z. J., C. B., X. J., W. C.-M. and L. Q.-Z. participated in the early development of the project. C. W.-D. and W. J., and F. C.-A. conceived and designed the project. C. W.-D. overall supervised the project. The manuscript was written through contributions of all authors. All authors have given approval to the final version of the manuscript.

## Conflicts of interest

The authors declare no competing interests.

## Supplementary Material

SC-OLF-D5SC00050E-s001
