# Supplementary material for: Facile synthesis of recyclable polythioimidocarbonates via aromatization-driven alternating copolymerization of para-quinone methide and isothiocyanates
Source: Chem Sci. 2025 Feb 21;16(13):5493–502. doi: 10.1039/d5sc00050e (PMC11866116; doi:10.1039/d5sc00050e)
Supplement: SC-016-D5SC00050E-s001 [file SC-016-D5SC00050E-s001.pdf]

Supplementary Information (SI) for Chemical Science.
This journal is © The Royal Society of Chemistry 2025

Supporting Information for

Facile Synthesis of Recyclable Polythioimidocarbonates *via* Aromatization-driven Alternating Copolymerization of *para*-Quinone Methide and Isothiocyanates

Wen-Dao Chu,*[a] Si-Yu Dan,[a] Jie Zhan,[a] Bo Chen,[a] Ji Xian,[b] Chun-Mei Wang,[a] Quan-Zhong Liu,[a] Jincai Wu,*[b] and Chun-An Fan*[b]

[a] Chemical Synthesis and Pollution Control Key Laboratory of Sichuan Province, College of Chemistry and Chemical Engineering, China West Normal University, Nanchong 637002, P.R. China.

[b]State Key Laboratory of Applied Organic Chemistry, College of Chemistry and Chemical Engineering, Lanzhou University, 222 Tianshui Nanlu, Lanzhou 730000, China

TABLE OF CONTENTS

Materials, Reagents, and Methods……………………………………………………………S2

Synthesis of *p*-QM……………………………………………………………………………S3

Procedure for polymerization and polymer characterizations……………………………..S5

Kinetic studies and control experiments…………………………………………………….S11

Thermal properties of polymers……………………………………………………………….S19

Chemical Recycling to Monomer……………………………………………………………...S20

DFT Study……………………………………………………………………………………..S21

References……………………………………………………………………………………..S61

Experimental Details

Materials, Reagents, and Methods

All syntheses and manipulations of air- and moisture-sensitive materials were performed under a dry nitrogen atmosphere in a glovebox or using standard Schlenk techniques. ^1H NMR and ^{13}C NMR spectra were recorded respectively at 400 MHz and 100 MHz on Bruker Avance 400M. ^1H NMR chemical shifts are reported in ppm relative to residual protons in deuterated solvent. The chemical shift for chloroform-d is δ 7.26 ppm. ^{13}C NMR chemical shifts are reported in ppm versus residual ^{13}C in the solvent: δ 77.0 ppm for chloroform-d. The molecular weights (M_n and M_w) and the molecular mass distributions (M_w/ M_n) of the polymer samples were measured by gel permeation chromatography (GPC) at 30 °C using THF as a solvent, an eluent flow rate of 1 mL/min, and narrow polystyrene standards as reference samples. The measurements were performed using a Waters 1525 system that was equipped with a Waters 2414 Refractive Index detector using WAT054405 Styragel columns. Matrix-Assisted Laser Desorption/Ionization Time of Flight (MALDI-TOF) Mass Spectrometry conditions were as follows. Instrument type: Bruker autoflex speed MALDI-TOF mass spectrometer, adjust to reflection mode, Power 80, P. Ext at 5000.00. The MALDI-TOF mass spectroscopic data were obtained using trans-2-[3-(4-tertbutylphenyl)-2-methyl-2-propenylidene]alonitrile (DCTB) as the matrix (10 mg/mL in THF), sodium trifluoroacetate as the cationization agent (10 mg/mL in THF) and samples were dissolved in THF (10 mg/mL). The solutions of samples, matrix and salt were mixed in a volume ratio of 1:1:1; then the mixed solution (1 μL) was hand-spotted on a stainless steel MALDI target, which allowed to be dried completely. Differential scanning calorimetry (DSC) measurements: using 10-15 mg of material, DSC experiments were performed on a DZ-DSC300C instrument with a heat (0-160 °C) at a heating rate of 20 °C/min and cooling rate of 10 °C/min under N_2 atmosphere. Thermal gravimetric analyzer (TGA): using 10-15 mg of material, TGA experiments were performed on a Linseis PT 1600 instrument, with a heat (25-500 °C) at a rate of 10 °C/min under N_2 atmosphere. Decomposition temperatures (Td, defined by the temperature of 5 % weight loss).

Toluene, THF, and hexane were dried by refluxing with sodium and benzophenone ketyl, the latter serves as an indicator. CH_2Cl_2 was distilled from CaH_2. 1,5,7-Triazabicyclo[4.4.0]dec-5-ene (TBD), 4-Dimethylaminopyridine (DMAP), *p*-methoxybenzoic acid, and isothiocyanate **1b-1c** were purchased from Energy Chemical

and purified via vacuum sublimation. Isothiocyanates **1a** and **1d** were distilled under reduced pressure before use. Phthalic acids **2b-2d** were dehydrated by stirring overnight at 100 °C under vacuum. *p*-QM was dehydrated by stirring overnight at 50 °C under vacuum.

Synthesis of *p*-QM

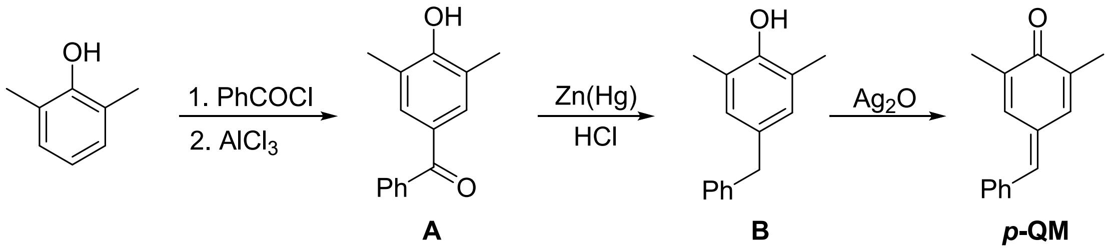

***p*-QM** was prepared according to literature procedures.[1] A mixture of 2,6-xylenol (6.1 g, 50 mmol) and benzoyl chloride (7.0 g, 50 mmol) was heated at 140 °C for 4 h with stirring. To the mixture was added 8.5 g (63 mmol) of aluminum chloride and then the mixture was stirred at 170 °C for 45 min. After the mixture cooled to 0 °C, aqueous hydrochloric acid (6 N) was added to quench the reaction, and the resulting mixture was extracted with CH_2Cl_2 (100 mL × 3). The extracts were combined, washed twice with brine, and dried over anhydrous magnesium sulfate. After the solvent evaporated, a residual solid was recrystallized from a mixed solution of ethyl acetate and hexane (1/5 v/v) to give 9.8 g (76%) of **A** as white plates. Zinc powder (58 g, 880 mmol) was added to a solution of mercuric chloride (0.8 g, 2.9 mmol) in 80 mL of water, and the mixture was stirred at room temperature for 30 min. The supernatant water was decanted off, and the precipitate was washed twice with water. Then, 80 mL of water and 60 mL of concentrated hydrochloric acid were added. To the suspension was added a solution of 18 g (78 mmol) of **A** in 100 mL of ethanol, and the mixture was refluxed for 8 h. After cooling to room temperature, the reaction mixture was extracted twice with diethyl ether. The extracts were combined, washed with brine, and dried over magnesium sulfate. After the diethyl ether evaporated, the residual pale yellow oil was distilled under reduced pressure to give 9.4 g (56%) of **B** as white needles. **B** (3.6 g, 17 mmol) was dissolved in 200 mL of diethyl ether, and to the solution was added 7.2 g (32 mmol) of silver oxide; the mixture was stirred at room temperature for 4 h. The reaction mixture was filtered to remove silver oxide, and then the diethyl ether was

evaporated. The residual solid was recrystallized from hexane to give 2.6 g (75%) of ***p*-QM** as yellow needles. **^{1}H NMR** (400 MHz, $CDCl_3$, ppm) δ 7.52 (s, 1H), 7.48 – 7.37 (m, 5H), 7.17 (s, 1H), 7.05 (s, 1H), 2.06 (dd, J = 3.1, 1.0 Hz, 6H). **^{13}C NMR** (100 MHz, $CDCl_3$, ppm) δ187.5, 142.8, 139.0, 137.8, 135.9, 135.8, 132.0, 131.5, 130.6, 129.4, 128.9, 17.0, 16.4.

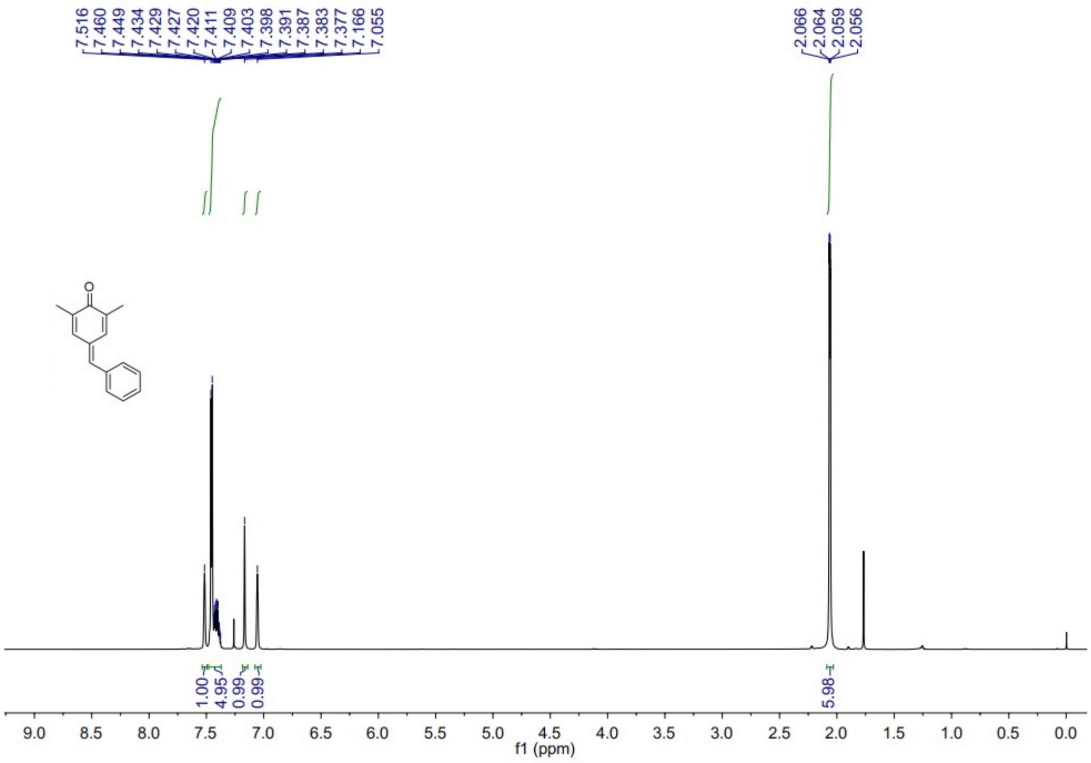

Figure S1. ^{1}H NMR ($CDCl_3$) spectrum of *p*-QM.

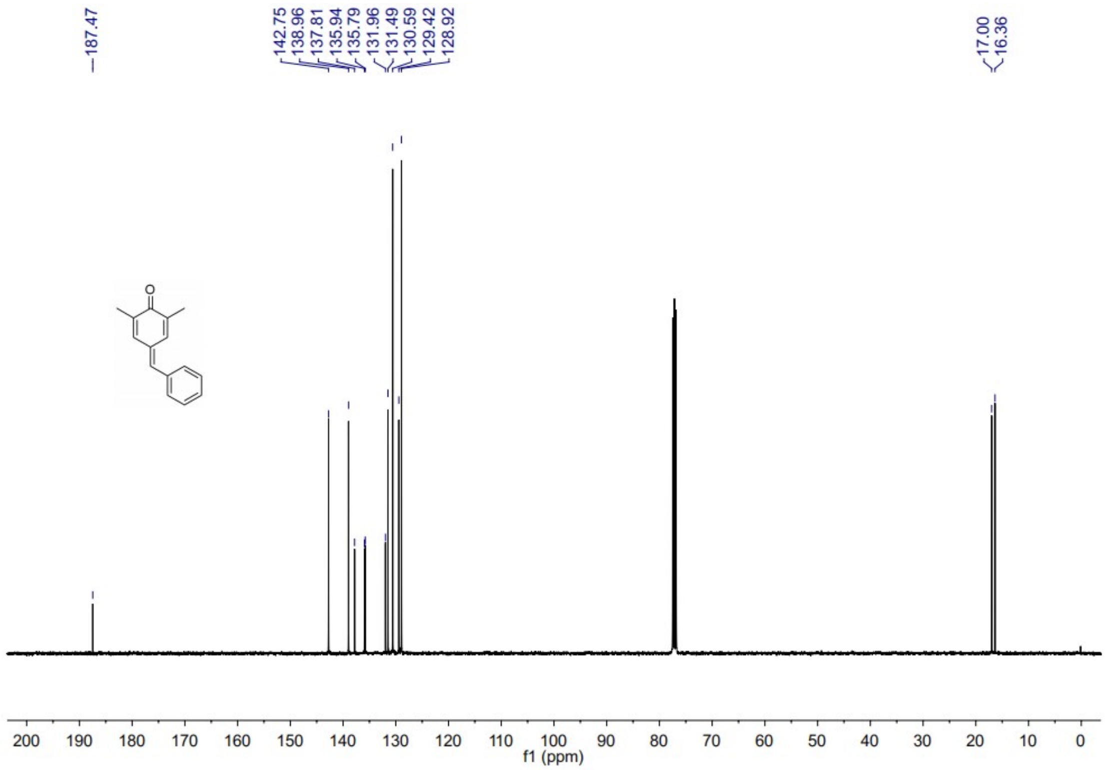

Figure S2. ^{13}C NMR ($CDCl_3$) spectrum of *p*-QM.

Procedure for polymerization

Polymerizations were performed in 10 mL glass reactors inside the glovebox. In a typical polymerization reaction, the reactor was charged with *p*-QM, isothiocyanate and THF. Acid catalyst was introduced into the mixture and stirred for 2 minutes. Then TBD was added to the resulting solution. The mixture was stirred at room temperature. After a desired period of time, an aliquot was withdrawn to determine *p*-QM conversion by ^{1}H NMR spectroscopy, and the remaining solution was quenched with a few drops of MeOH. The polymer was precipitated by adding hexane (10 mL). Polymer was obtained by recrystallization from a CH_2Cl_2/hexane mixed solvent and dried in a vacuum oven at 60 °C to a constant weight.

Table S1. Raw data over equilibrium conversion at various ratios of isothiocyanate[a]

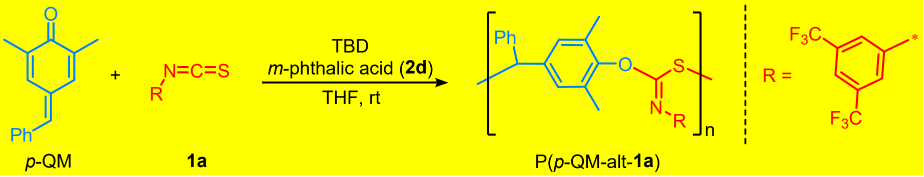

entry	feeding	time (h)	conv. (%)[b]	$M_{n,\text{theo}}$ (kDa)[c]	$M_{n,\text{GPC}}$ (kDa)[d]	$Đ$[d]
1	p-QM/**1a**/TBD/**2d** (100/100/1/0.5)	1.0	70	33.8	38.1	1.23
2	p-QM/**1a**/TBD/**2d** (100/200/1/0.5)	1.0	82	39.6	45.4	1.24
3	p-QM/**1a**/TBD/**2d** (100/300/1/0.5)	1.0	90	43.5	48.6	1.24
4	p-QM/**1a**/TBD/**2d** (100/400/1/0.5)	1.0	97	46.8	51.2	1.23

[a]The copolymerization was conducted in THF in a glovebox at 25 °C, $[p\text{-QM}]_0$ = 0.5 M. [b]Conversion of p-QM, determined by ^{1}H NMR spectroscopy. [c]Calculated molar mass based on $[p\text{-QM}]_0/[\text{TBD}]_0$ ratio and conversion. [d]M_n and $Đ$ were determined by GPC analysis in THF.

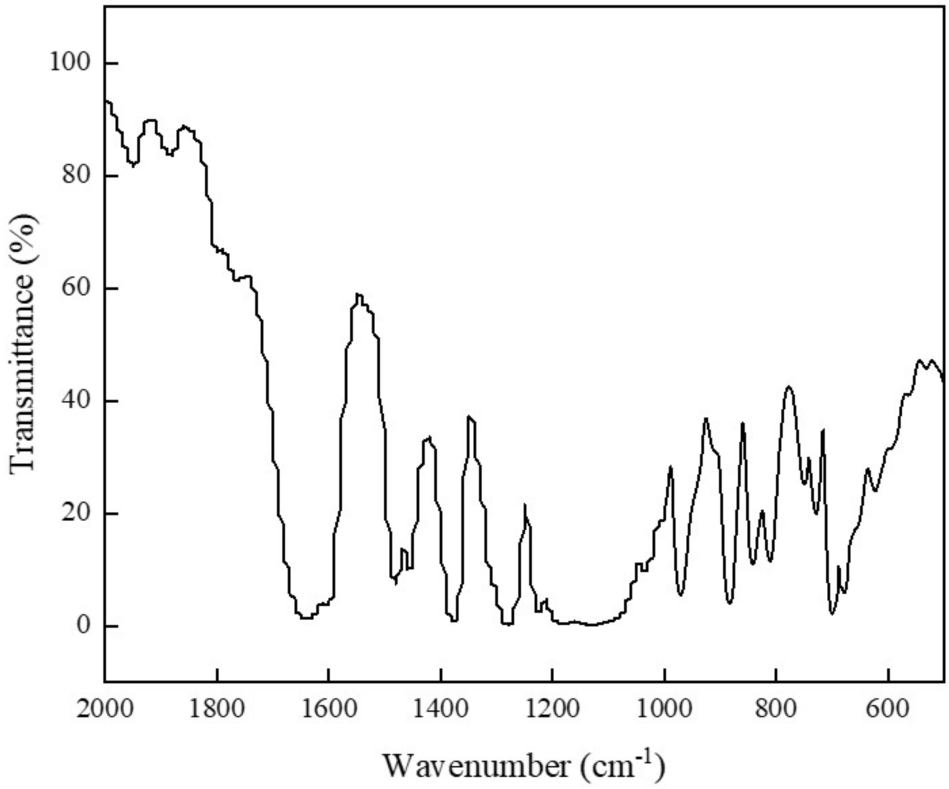

Figure S3. Fourier-transform infrared (FTIR) spectrum of P(p-QM-alt-**1a**)

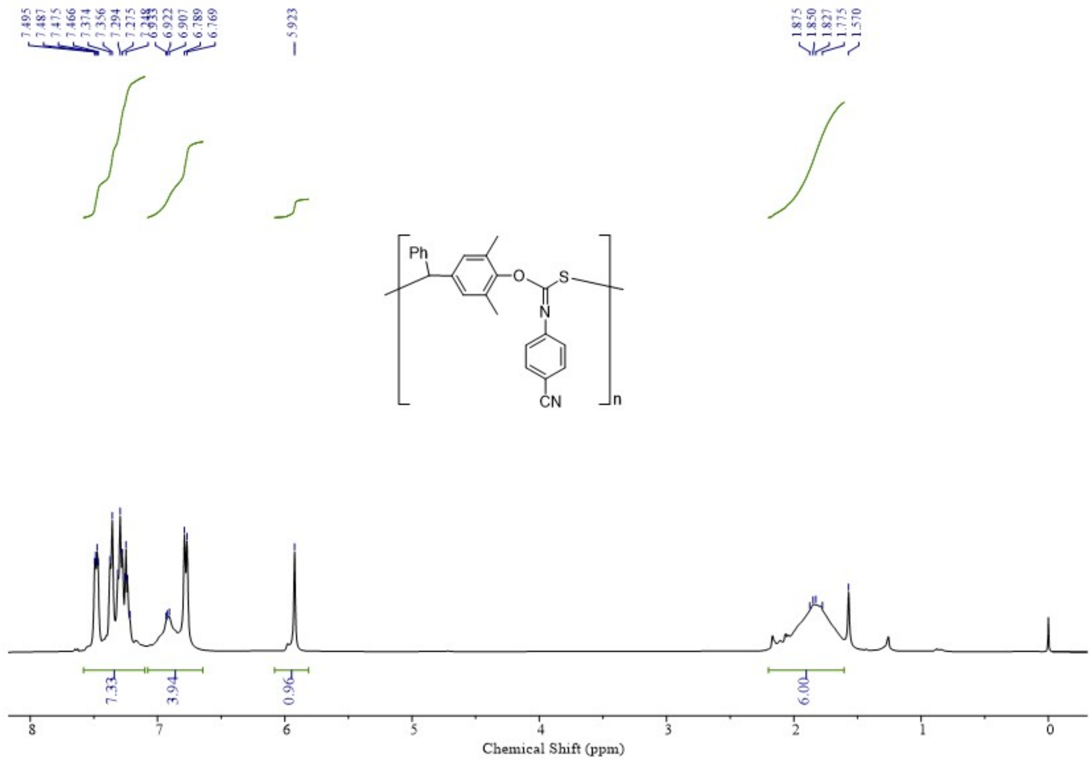

Figure S4. ^{1}H NMR ($CDCl_3$) spectrum of P(*p*-QM-alt-**1b**) obtained by [*p*-QM]/[**1b**] /[TBD]/[**2d**] = 100/400/1/0.5

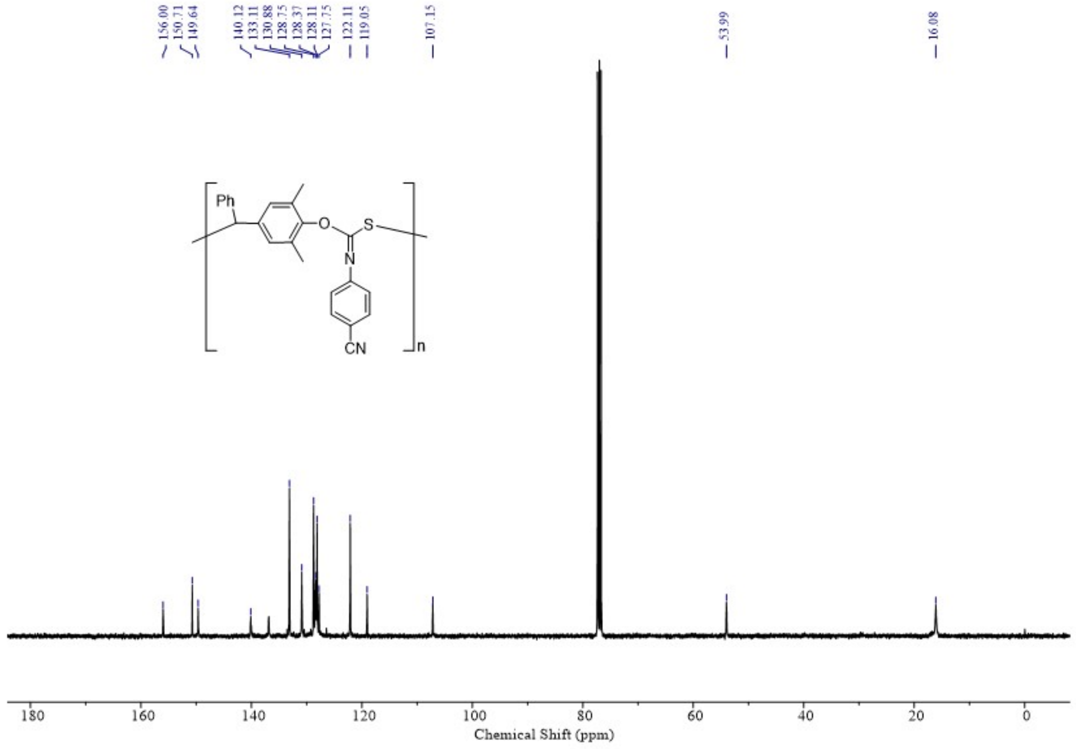

Figure S5. ^{13}C NMR ($CDCl_3$) spectrum of P(*p*-QM-alt-**1b**) obtained by [*p*-QM]/[**1b**] /[TBD]/[**2d**] = 100/400/1/0.5

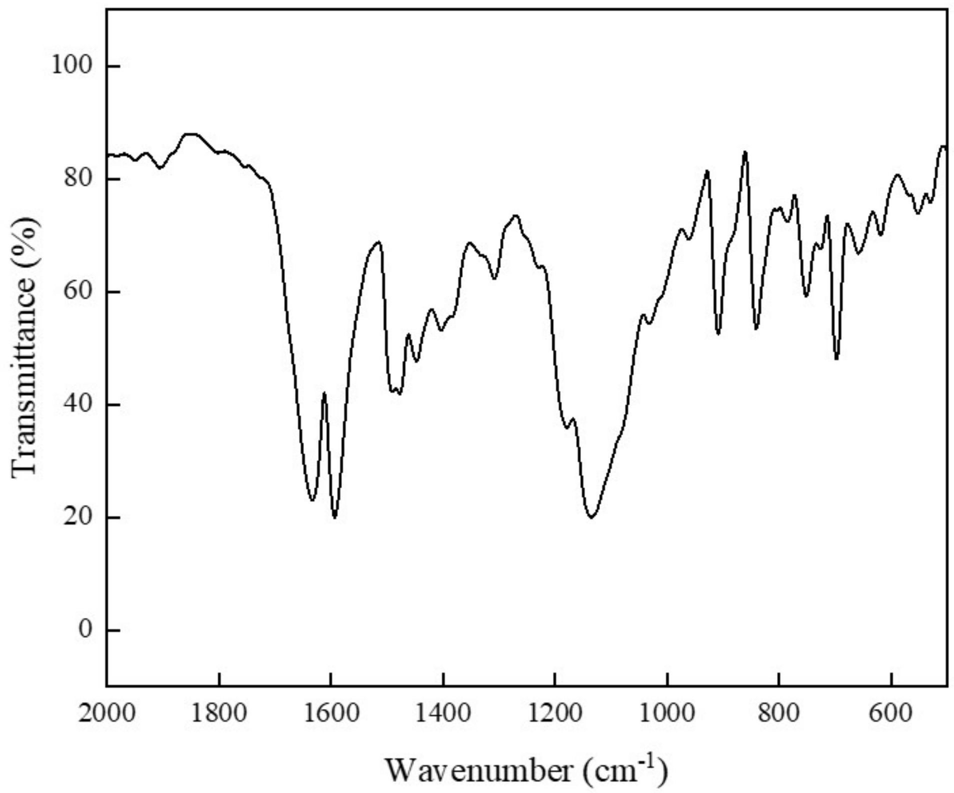

Figure S6. Fourier-transform infrared (FTIR) spectrum of P(*p*-QM-alt-**1b**)

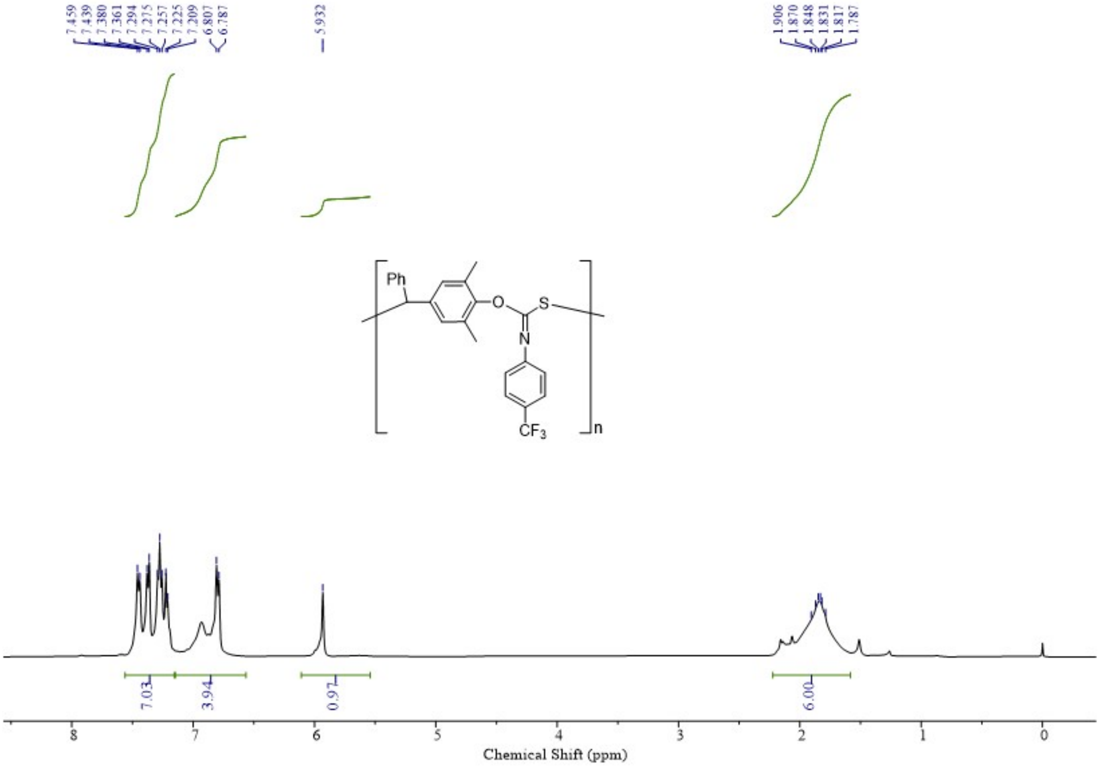

Figure S7. ^{1}H NMR ($CDCl_3$) spectrum of P(*p*-QM-alt-**1c**) obtained by [*p*-QM]/[**1c**]/[TBD]/[**2d**] = 100/400/1/0.5

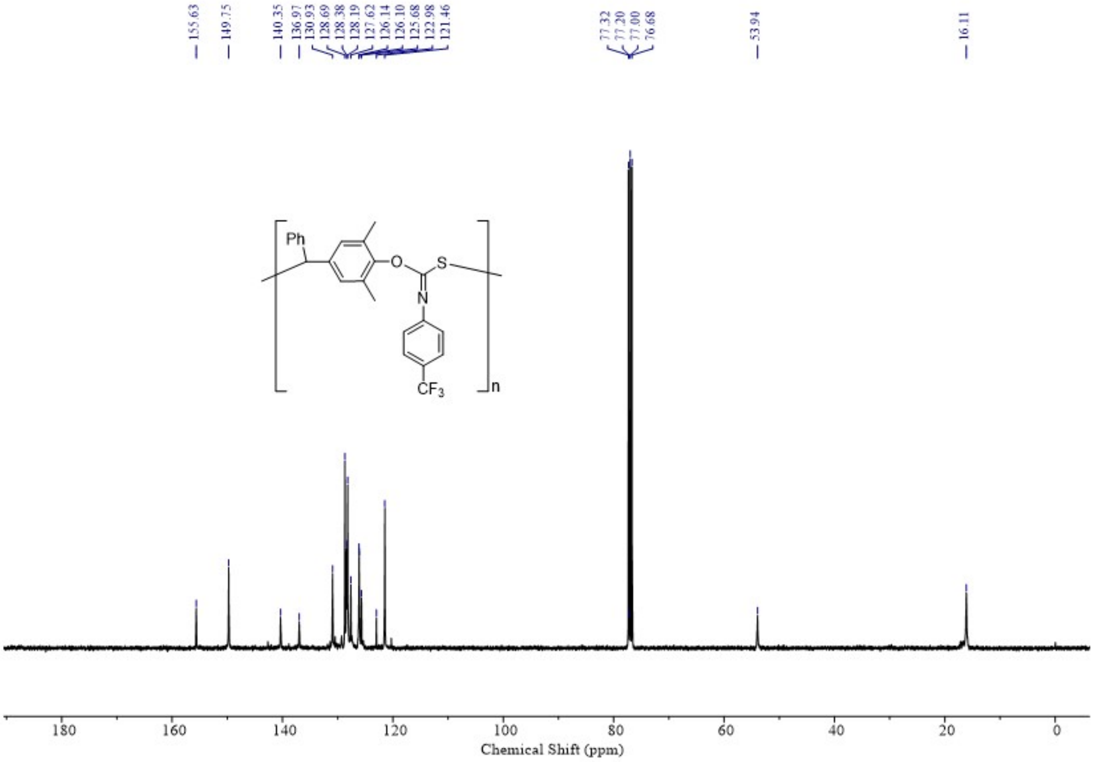

Figure S8. ^{13}C NMR ($CDCl_3$) spectrum of P(*p*-QM-alt-**1c**) obtained by [*p*-QM]/[**1c**] /[TBD]/[**2d**] = 100/400/1/0.5

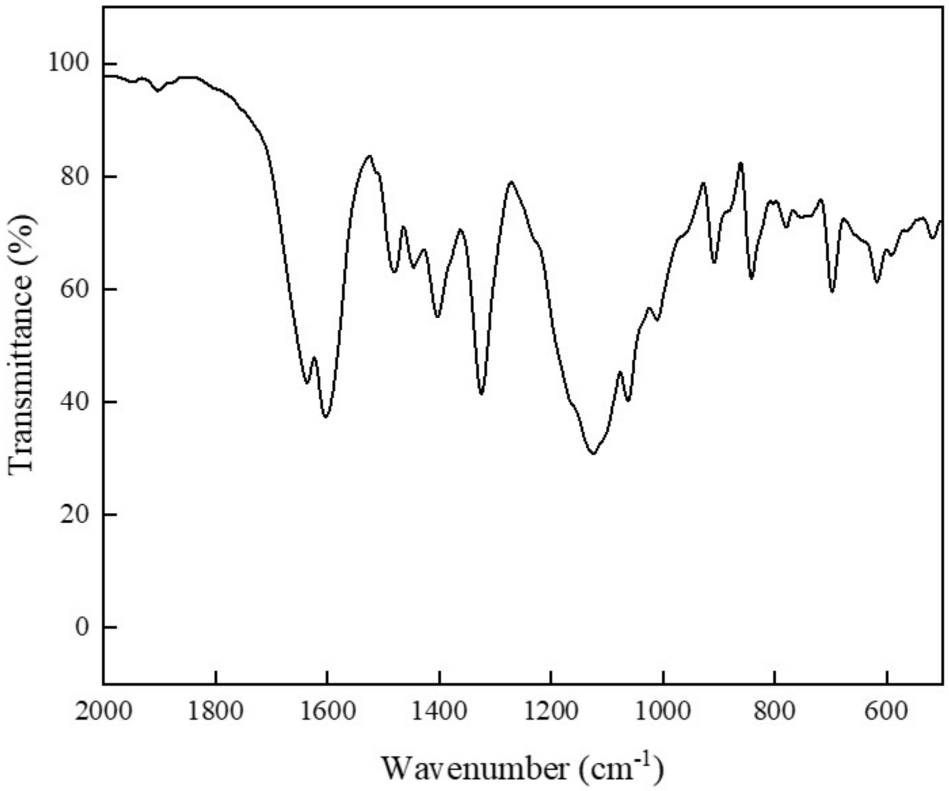

Figure S9. Fourier-transform infrared (FTIR) spectrum of P(*p*-QM-alt-**1c**)

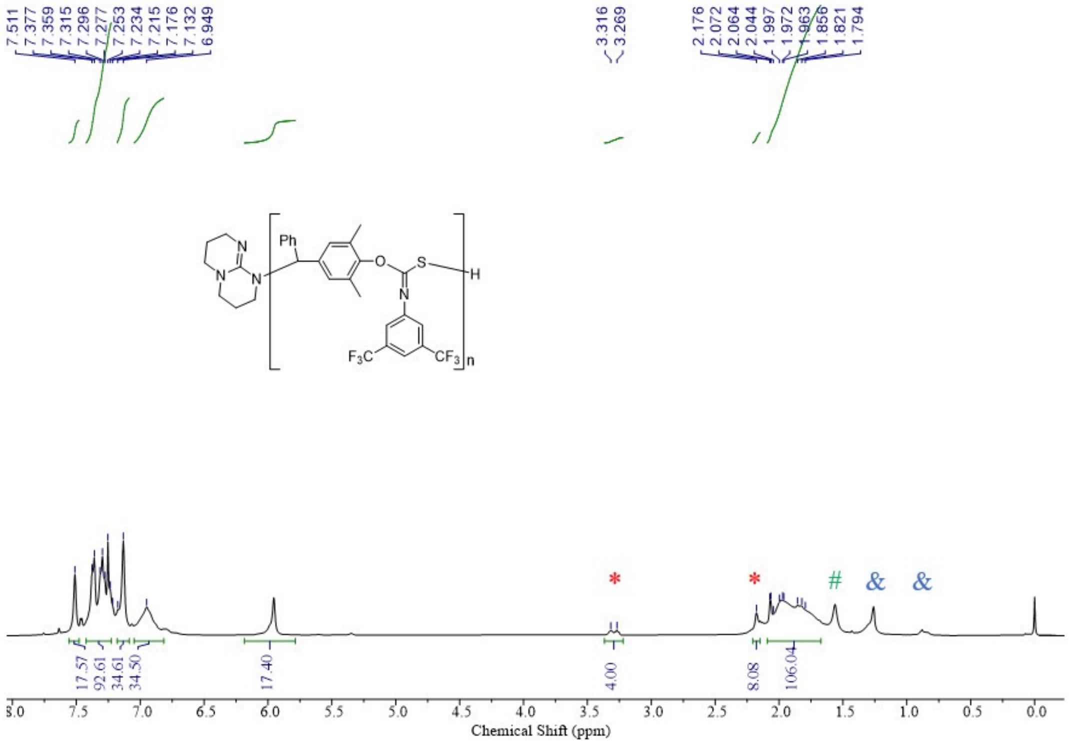

Figure S10. ^1H NMR ($CDCl_3$) spectrum of P(*p*-QM-alt-**1a**) obtained by [*p*-QM]/[**1a**]/[TBD]/[**2d**] = 15/60/1/0.5. (*TBD, # H_2O, & *n*-hexane)

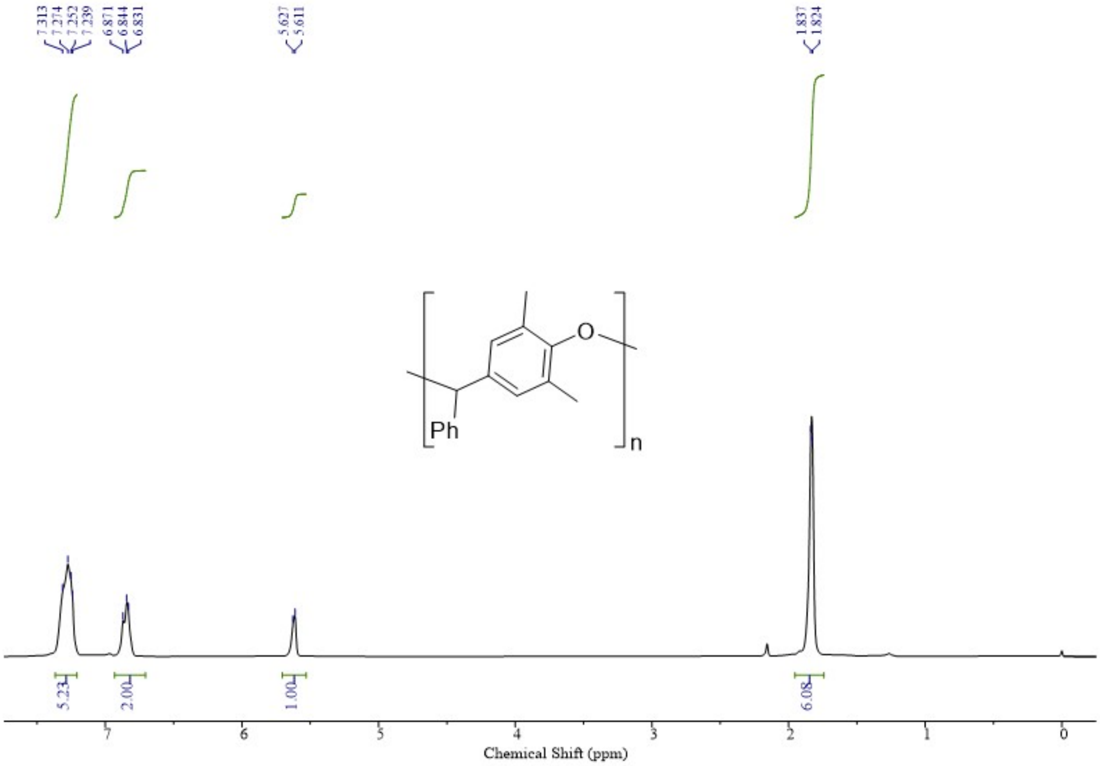

Figure S11. ^1H NMR ($CDCl_3$) spectrum of *p*-QM homopolymerization.

Typical procedure of kinetic studies

In a glovebox, a mixture of *p*-QM (52.5 mg, 0.25 mmol), isothiocyanate **1a** (182 μL, 271 mg, 1.0 mmol), acid catalyst **2d** (0.21 mg, 0.00125 mmol) and 0.4 mL of THF-d^8 was added to a J Youngs tap NMR tube. Then, a 0.1 mL solution of TBD in THF-d^8 (0.35 mg, 0.0025 mmol) was added to the resulting solution. The reaction took place at room temperature. ^{1}H NMR spectroscopic studies were used to analyze the *p*-QM conversion.

Other kinetic studies with different equivalents of **1a**, acid or initiator were carried out in a similar manner.

Table S2. Kinetic studies of *p*-QM/**1a** copolymerization at fixed *p*-QM-to-catalyst ratio ($[\mathbf{2d}]_0/[\text{TBD}]_0/[p\text{-QM}]_0/[\mathbf{1a}]_0$ = 1:2:200:800)[a]

Entry	$[\mathbf{2d}]_0/[\text{TBD}]_0/[p\text{-QM}]_0/[\mathbf{1a}]_0$	Time (min)	% *p*-QM Conv.[b]	$ln([p\text{-QM}]_0/[p\text{-QM}]_t)$
S1	1:2:200:800	12	21	0.236
S2	1:2:200:800	16	34	0.416
S3	1:2:200:800	20	45	0.598
S4	1:2:200:800	23	55	0.799
S5	1:2:200:800	27	62	0.968
S6	1:2:200:800	30	71	1.238
S7	1:2:200:800	35	77	1.470
S8	1:2:200:800	39	82	1.715
S9	1:2:200:800	44	86	1.966
S10	1:2:200:800	48	88	2.120
S11	1:2:200:800	52	90	2.302
S12	1:2:200:800	56	92	2.526

Copolymerization conditions: 0.00125 mmol catalyst **2d**, 0.5 mL THF-d^8, rt. [a]All runs showed excellent copolymerization selectivity based on ^{1}H NMR determination. [b]Based on ^{1}H NMR analysis of the reaction mixture.

Table S3. Kinetic studies of *p*-QM/**1a** copolymerization at different **1a**-to-catalyst ratio[a]

Entry	[**2d**]$_0$/[TBD]$_0$/[*p*-QM]$_0$/[**1a**]$_0$	Time (min)	% *p*-QM Conv.[b]	*ln*([*p*-QM]$_0$/[*p*-QM]$_t$)
S1	1:2:200:600	6	6	0.062
S2	1:2:200:600	10	19	0.211
S3	1:2:200:600	14	36	0.446
S4	1:2:200:600	19	55	0.799
S5	1:2:200:600	23	68	1.139
S6	1:2:200:600	27	75	1.386
S7	1:2:200:600	31	81	1.661
S8	1:2:200:600	36	88	2.120
S9	1:2:200:600	40	90	2.303
S10	1:2:200:600	44	92	2.526
S11	1:2:200:600	48	93	2.659
S12	1:2:200:600	52	94	2.813
S13	1:2:200:800	12	21	0.236
S14	1:2:200:800	16	34	0.416
S15	1:2:200:800	20	45	0.598
S16	1:2:200:800	23	55	0.799
S17	1:2:200:800	27	62	0.968
S18	1:2:200:800	30	71	1.238
S19	1:2:200:800	35	77	1.470
S20	1:2:200:800	39	82	1.715
S21	1:2:200:800	44	86	1.966
S22	1:2:200:800	48	88	2.120
S23	1:2:200:800	52	90	2.302
S24	1:2:200:800	56	92	2.526
S25	1:2:200:1000	11	24	0.274

S26	1:2:200:1000	16	42	0.545
S27	1:2:200:1000	21	58	0.868
S28	1:2:200:1000	24	66	1.079
S29	1:2:200:1000	28	73	1.309
S30	1:2:200:1000	32	79	1.561
S31	1:2:200:1000	35	83	1.772
S32	1:2:200:1000	39	86	1.966
S33	1:2:200:1000	44	90	2.303
S34	1:2:200:1000	49	92	2.526
S35	1:2:200:1200	9	22	0.248
S36	1:2:200:1200	13	41	0.528
S37	1:2:200:1200	17	55	0.799
S38	1:2:200:1200	21	66	1.079
S39	1:2:200:1200	26	75	1.386
S40	1:2:200:1200	31	82	1.715
S41	1:2:200:1200	38	88	2.120
S42	1:2:200:1200	46	92	2.526

Copolymerization conditions: 0.25 mmol *p*-QM, 0.5 mL THF-d^8, rt. [a]All runs showed excellent copolymerization selectivity based on ^{1}H NMR determination. [b]Based on ^{1}H NMR analysis of the reaction mixture.

Table S4. Kinetic studies of *p*-QM/**1a** copolymerization at different **1a**-to-catalyst ratio

Entry	[**2d**]$_0$/[TBD]$_0$/[*p*-QM]$_0$/[**1a**]$_0$	Observed rate coefficient, k_{obs} (min^{-1})[a]	ln(k_{obs})	Concentrations [**1a**] (M)	ln[**1a**]
S1	1:2:200:600	0.064	-2.742	1.5	0.405
S2	1:2:200:800	0.053	-2.936	2	0.693
S3	1:2:200:1000	0.061	-2.797	2.5	0.916
S4	1:2:200:1200	0.062	-2.781	3	1.099

[a]Calculated from the slope of the fitted regression line of Table S2.

Table S5. Kinetic studies of *p*-QM/**1a** copolymerization at different monomer-to-catalyst ratio[a]

Entry	[**2d**]$_0$/[TBD]$_0$/[*p*-QM]$_0$/[**1a**]$_0$	Time (min)	% *p*-QM Conv.[b]	*ln*([*p*-QM]$_0$/[*p*-QM]$_t$)
S1	0.25:2:200:800	5	13	0.139
S2	0.25:2:200:800	9	28	0.329
S3	0.25:2:200:800	14	66	1.079
S4	0.25:2:200:800	24	77	1.470
S5	0.25:2:200:800	28	84	1.833
S6	0.25:2:200:800	32	89	2.207
S7	0.25:2:200:800	36	91	2.408
S8	0.25:2:200:800	39	93	2.659
S9	0.25:2:200:800	43	94	2.813
S10	0.25:2:200:800	50	96	3.219
S11	0.5:2:200:800	12	37	0.462
S12	0.5:2:200:800	19	56	0.821
S13	0.5:2:200:800	25	72	1.273
S14	0.5:2:200:800	29	80	1.609
S15	0.5:2:200:800	33	85	1.897
S16	0.5:2:200:800	38	91	2.408
S17	0.5:2:200:800	42	92	2.526
S18	0.5:2:200:800	52	95	2.996
S19	1:2:200:800	12	21	0.236
S20	1:2:200:800	16	34	0.416
S21	1:2:200:800	20	45	0.598
S22	1:2:200:800	23	55	0.799
S23	1:2:200:800	27	62	0.968
S24	1:2:200:800	30	71	1.238
S25	1:2:200:800	35	77	1.470
S26	1:2:200:800	39	82	1.715

S27	1:2:200:800	44	86	1.966
S28	1:2:200:800	48	88	2.120
S29	1:2:200:800	52	90	2.303
S30	1:2:200:800	56	92	2.526
S31	2:2:200:800	32	3	0.030
S32	2:2:200:800	39	5	0.051
S33	2:2:200:800	44	6	0.062
S34	2:2:200:800	54	14	0.151
S35	2:2:200:800	58	21	0.236
S36	2:2:200:800	62	28	0.329
S37	2:2:200:800	66	41	0.528
S38	2:2:200:800	70	51	0.713
S39	2:2:200:800	74	61	0.942
S40	2:2:200:800	77	70	1.204
S41	2:2:200:800	82	76	1.427
S42	2:2:200:800	85	83	1.772
S43	2:2:200:800	89	86	1.966
S44	2:2:200:800	93	89	2.207
S45	2:2:200:800	97	92	2.526
S46	2:2:200:800	101	93	2.659
S47	2:2:200:800	105	93	2.659
S48	2:2:200:800	108	94	2.813
S49	2:2:200:800	112	95	2.995
S50	2:2:200:800	116	95	2.995

Copolymerization conditions: 0.25 mmol *p*-QM, 0.5 mL THF-d^8, rt. [a]All runs showed excellent copolymerization selectivity based on ^{1}H NMR determination. [b]Based on ^{1}H NMR analysis of the reaction mixture.

Table S6. Kinetic studies of *p*-QM/**1a** copolymerization at different monomer-to-catalyst ratio

Entry	[**2d**]$_0$/[TBD]$_0$/[*p*-QM]$_0$/[**1a**]$_0$	Observed rate coefficient, *k*obs (min^{-1})[a]	ln(*k*obs)	Concentrations [**2d**] (M)	ln[**2d**]
S1	0.25:2:200:800	0.070	-2.666	6.25×10^{-4}	-7.378
S2	0.5:2:200:800	0.068	-2.694	1.25×10^{-3}	-6.685
S3	1:2:200:800	0.053	-2.936	2.5×10^{-3}	-5.991
S4	2:2:200:800	0.043	-3.135	5×10^{-3}	-5.298

[a]Calculated from the slope of the fitted regression line of Table S4.

Table S7. Kinetic studies of *p*-QM/**1a** copolymerization at different monomer-to-initiator ratio[a]

Entry	[**2d**]$_0$/[TBD]$_0$/[*p*-QM]$_0$/[**1a**]$_0$	Time (min)	% *p*-QM Conv.[b]	*ln*([*p*-QM]$_0$/[*p*-QM]$_t$)
S1	1:1.2:200:800	28	40	0.511
S2	1:1.2:200:800	34	59	0.892
S3	1:1.2:200:800	40	70	1.204
S4	1:1.2:200:800	44	76	1.427
S5	1:1.2:200:800	50	80	1.609
S6	1:1.2:200:800	55	85	1.897
S7	1:1.2:200:800	60	86	1.966
S8	1:1.2:200:800	65	87	2.040
S9	1:1.2:200:800	71	89	2.207
S10	1:1.2:200:800	76	90	2.303
S11	1:1.2:200:800	85	92	2.526
S12	1:1.2:200:800	92	93	2.659
S13	1:1.5:200:800	16	13	0.139
S14	1:1.5:200:800	20	28	0.329
S15	1:1.5:200:800	25	42	0.545
S16	1:1.5:200:800	29	51	0.713

S17	1:1.5:200:800	33	59	0.892
S18	1:1.5:200:800	36	64	1.022
S19	1:1.5:200:800	40	69	1.171
S20	1:1.5:200:800	44	74	1.347
S21	1:1.5:200:800	49	78	1.514
S22	1:1.5:200:800	57	84	1.833
S23	1:2:200:800	12	21	0.236
S24	1:2:200:800	16	34	0.416
S25	1:2:200:800	20	45	0.598
S26	1:2:200:800	23	55	0.799
S27	1:2:200:800	27	62	0.968
S28	1:2:200:800	30	71	1.238
S29	1:2:200:800	35	77	1.470
S30	1:2:200:800	39	82	1.715
S31	1:2:200:800	44	86	1.966
S32	1:2:200:800	48	88	2.120
S33	1:2:200:800	52	90	2.302
S34	1:2:200:800	56	92	2.526
S35	1:2.5:200:800	8	15	0.163
S36	1:2.5:200:800	12	33	0.400
S37	1:2.5:200:800	16	50	0.693
S38	1:2.5:200:800	21	68	1.139
S39	1:2.5:200:800	26	80	1.609
S40	1:2.5:200:800	31	86	1.966
S41	1:2.5:200:800	36	91	2.408

Copolymerization conditions: 0.25 mmol *p*-QM, 0.5 mL THF-d^8, rt. [a]All runs showed excellent copolymerization selectivity based on ^{1}H NMR determination. [b]Based on ^{1}H NMR analysis of the reaction mixture.

Table S8. Kinetic studies of *p*-QM/**1a** copolymerization at different monomer-to-initiator ratio

Entry	[**2d**]$_0$/[TBD]$_0$/[*p*-QM]$_0$/[**1a**]$_0$	Observed rate coefficient, *k*obs (min^{-1})[a]	ln(*k*obs)	Concentrations [TBD] (M)[*b*]	ln[TBD]
S1	1:1.2:200:800	0.032	-3.449	3×10^{-3}	-5.809
S2	1:1.5:200:800	0.041	-3.189	3.75×10^{-3}	-5.586
S3	1:2:200:800	0.053	-2.936	5×10^{-3}	-5.298
S4	1:2.5:200:800	0.082	-2.507	6.25×10^{-3}	-5.075

[a]Calculated from the slope of the fitted regression line of Table S6.

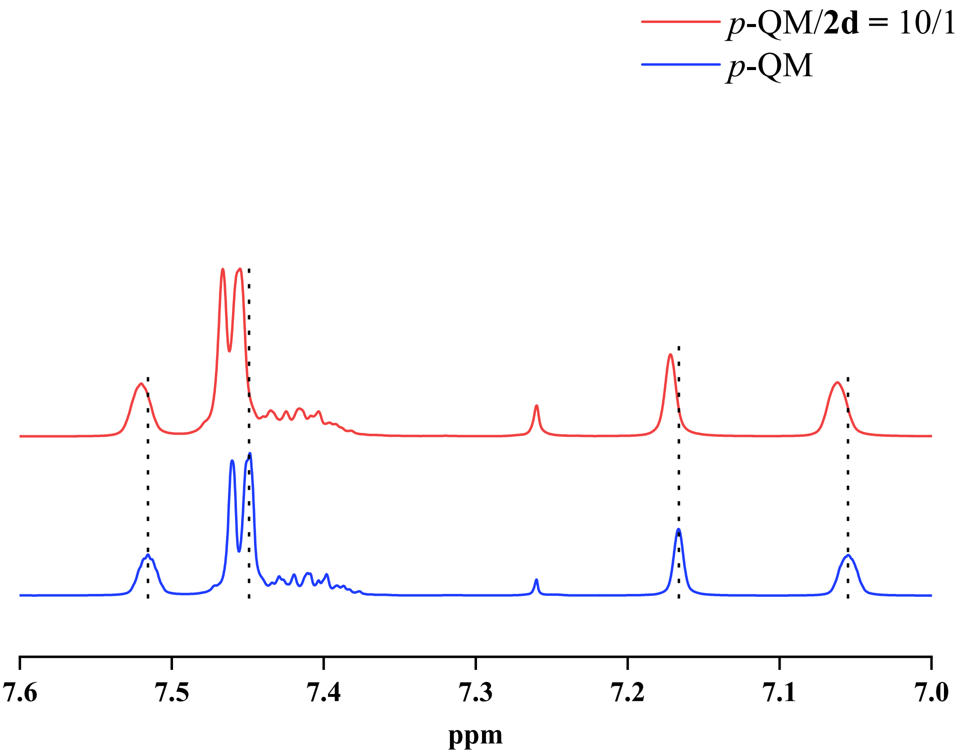

Figure S12. ^{1}H NMR studies of *p*-QM activated by **2c**.

Table S9. Studies of *p*-QM/**1a** copolymerization catalyzed by different acids at fixed *p*-QM-to-catalyst ratio ([acid]$_0$/[TBD]$_0$/[*p*-QM]$_0$/[**1a**]$_0$ = 1:1:100:400)

entry	acid	time (h)	conv. (%)	$M_{n,theo}$ (kDa)	$M_{n,GPC}$ (kDa)	PDI
1	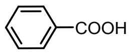	0.8	86	41.5	51.0	1.48
2	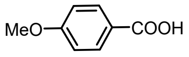	0.8	91	43.9	54.7	1.41

3	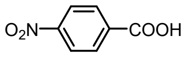	0.8	87	42.0	44.7	1.49
4	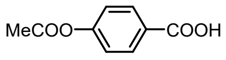	0.8	89	43.0	53.0	1.44
5	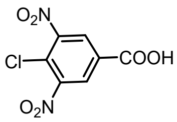	19	58	28.1	28.6	1.40
6	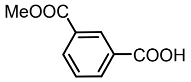	0.5	99	47.8	45.5	1.55
7	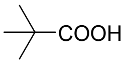	1	99	47.8	33.1	1.49
8	HCOOH	4	99	47.8	27.8	1.44

Copolymerization conditions: *p*-QM (52.5 mg, 0.25 mmol), isothiocyanate **1a** (182 μL, 271 mg, 1.0 mmol), TBD (0.0025 mmol), acid (0.0025 mmol), 0.5 mL THF, rt.

Thermal Properties of Polythioimidocarbonates

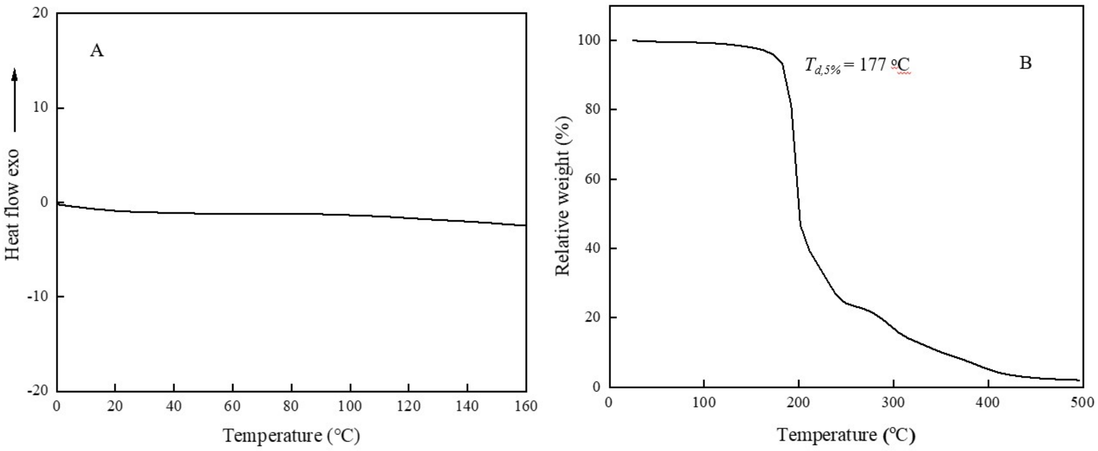

Figure S13. DSC (A) and TGA (B) curves of P(*p*-QM-alt-**1a**).

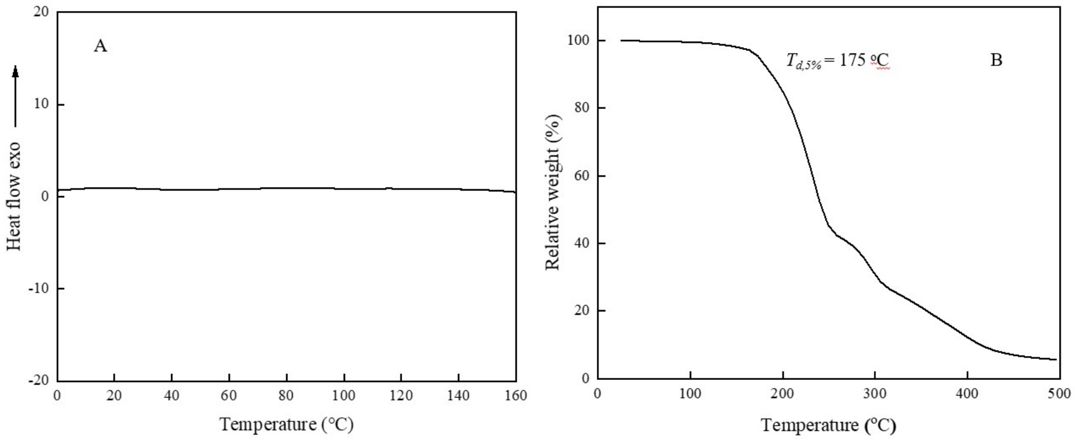

Figure S14. DSC (A) and TGA (B) curves of P(*p*-QM-alt-**1b**).

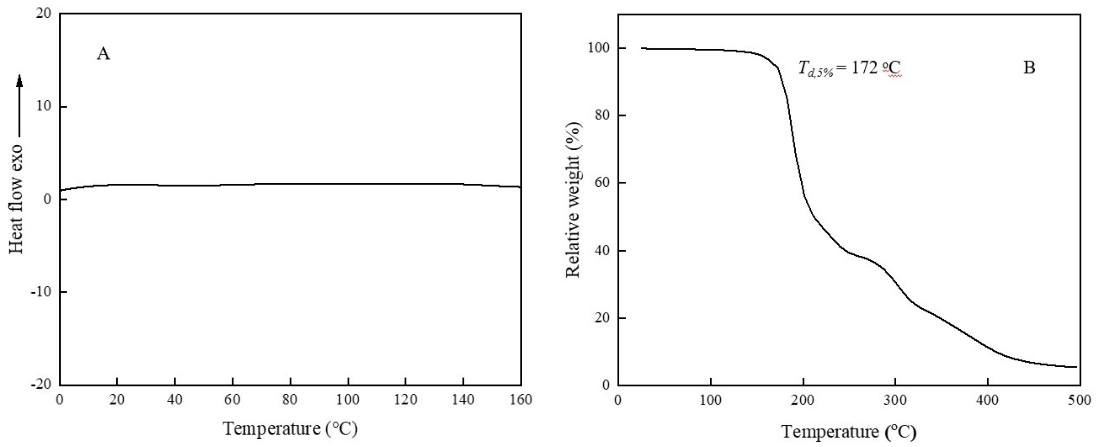

Figure S15. DSC (A) and TGA (B) curves of P(*p*-QM-alt-**1c**).

Bulk Thermal Depolymeriation of P(*p*-QM-alt-1a) and Monomers Recovery

Thermal depolymerization of **P(*p*-QM-alt-1a)** and recovery of monomers were caried out on the following experimental set-up. **P(*p*-QM-alt-1a)** (100 mg) were added into a sublimation device and kept at 190 ºC with stirring in vacuo for 2 minutes. When the coldfinger was fully covered with yellow sublimate, the reaction was stoped. Then the sublimate was collected, weighted, and characterized by NMR.

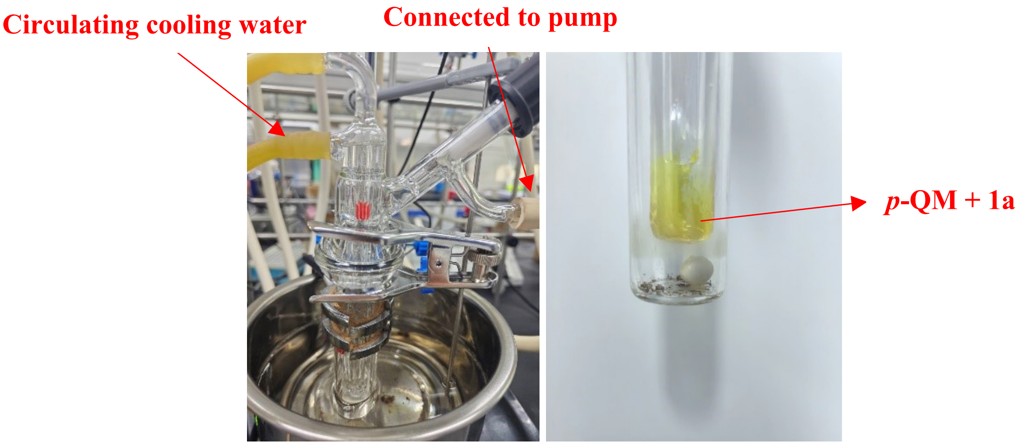

Figure S16. Thermal depolymerization of **P(*p*-QM-alt-1a)** and recovery of monomers via sublimation.

DFT Study

Computational Methods

The potential energy surface (PES) calculations were performed using Gaussian 16 C.02[2] and ORCA 6.0.1[3] software packages. Geometry optimization and harmonic frequency calculations were carried out with Gaussian 16 C.02 using the B3LYP[4] functional, def2-SVP[5] basis set, and the D3BJ[6] empirical dispersion correction. Transition states were verified by frequency analysis, ensuring the presence of a single imaginary frequency corresponding to the reaction coordinate, while all stable states exhibited no imaginary frequencies. Intrinsic reaction coordinate (IRC)[7] calculations were conducted to confirm the connectivity of each transition state to its corresponding reactants and products. Single-point energy calculations in THF solution were performed using ORCA 6.0.1 with the PWPB95[8] functional, def2-TZVPPD[9] basis set, and the D4[10] empirical dispersion correction. Solvent effects were modeled with the SMD[11] implicit solvation model for THF. The Gibbs free energy in THF solution was obtained by combining the following components: Gibbs free energy corrections from Gaussian 16 frequency analysis, single-point electronic energies from ORCA calculations in solution, and a correction for the gas-phase to solution free energy change (1.89 kcal/mol).

Molecular Coordinates

p-QM

Charge: 0 Multiplicity: 1

C	-3.12794100	-1.22982800	0.80034500
C	-1.90051800	-0.93069300	1.58218100
C	-0.67730600	-1.06337000	1.00969200
C	-0.50727500	-1.49051300	-0.37001800
C	-1.71613900	-1.70899900	-1.15077800
C	-2.96161500	-1.60840900	-0.62436400
H	0.21122700	-0.80113100	1.58521100
H	-1.59087400	-1.99464100	-2.19997200

C	-2.10927900	-0.46678500	2.99078300
H	-2.72775800	0.44522300	3.01062700
H	-2.67212600	-1.22095400	3.56420700
H	-1.15477500	-0.26536700	3.49762700
C	-4.21874300	-1.86180500	-1.39823900
H	-4.80388300	-2.66993900	-0.93024200
H	-4.86949700	-0.97258600	-1.38018700
H	-4.00532100	-2.13077700	-2.44260400
C	0.70791700	-1.66885400	-0.98142600
H	0.67606700	-1.85538200	-2.06087300
C	2.05368600	-1.62573100	-0.41699900
C	3.11729700	-1.20034100	-1.24272300
C	2.36111300	-2.03578200	0.89867000
C	4.42212800	-1.13352500	-0.75981900
H	2.90312900	-0.90419500	-2.27283300
C	3.66945900	-1.97780200	1.37660400
H	1.57465000	-2.44290800	1.53418200
C	4.70336800	-1.51681800	0.55566200
H	5.22609000	-0.78674700	-1.41325800
H	3.88666200	-2.30866200	2.39497900
H	5.72719200	-1.47271600	0.93409500
O	-4.24175600	-1.14137400	1.31032100

TBD

Charge: 0 Multiplicity: 1

C	-1.41530800	1.94786700	-2.22758400
C	-1.42556400	2.93406600	-1.06765700
C	0.38326100	1.56671500	-0.18518300
C	0.02891100	1.53626700	-2.52081200
H	-0.95850400	3.89344700	-1.37817000
H	-2.45766400	3.17063700	-0.75818900
H	-2.00548700	1.05992800	-1.94474000
H	-1.89317500	2.39605300	-3.11354100
H	0.54828600	2.36739900	-3.04053600
H	0.04038400	0.69398300	-3.23555200
C	-0.86286100	3.09848800	1.32364100
C	-0.31837900	2.29960300	2.49968400
C	1.09909900	1.84598300	2.17971800
H	-0.33497700	2.91591000	3.41180200
H	-0.33751200	4.07738500	1.26449200
H	-1.93103200	3.32839100	1.47553100
H	1.76932900	2.73278800	2.14473500
H	1.48461000	1.18495000	2.97209800
H	-0.95268100	1.41480700	2.67027400
N	-0.73200900	2.36461900	0.07605600
N	0.77472200	1.16211300	-1.34050100
N	1.07600100	1.11452700	0.93095500
H	1.92453600	0.63584500	0.64980200

***m*-phthalic acid**

Charge: 0 Multiplicity: 1

C	-3.06624000	0.62850900	-0.06663000
C	-1.66890000	0.58766300	-0.03636100
C	-0.93936000	1.77140800	0.12864700
C	-1.61071400	2.99614300	0.26330600
C	-3.00658900	3.03427400	0.23280000
C	-3.73367500	1.85610700	0.06853800
H	-1.13363500	-0.35575100	-0.13964600
H	-1.03220500	3.91149200	0.39125200
H	-3.52808700	3.98799600	0.33772200
H	-4.82474200	1.85942800	0.04141900
C	0.54796200	1.67553100	0.15428300
C	-3.89020700	-0.60200200	-0.23977100
O	-5.09836900	-0.61790900	-0.27227100
O	1.17206900	0.64706300	0.04255700
O	1.14889800	2.87267400	0.31775300
H	2.10569100	2.70224500	0.32063600
O	-3.14634600	-1.71920700	-0.35930100
H	-3.76926600	-2.45734300	-0.46660900

PHO

Charge: -1 Multiplicity: 1

C	1.11403500	-2.93314100	-0.52187300
C	-0.17693900	-2.41359800	-0.46682300
C	-0.54070400	-1.22040600	-1.20928200

C	0.53058000	-0.62573500	-1.98843600
C	1.80537400	-1.18541300	-2.00914000
C	2.13977600	-2.34686800	-1.28706000
H	1.34115600	-3.83932800	0.05874400
H	2.58372000	-0.69778600	-2.61440000
O	-1.70576500	-0.72809100	-1.17295400
C	-1.26579300	-3.03681500	0.35946700
H	-1.66872900	-2.31538000	1.09462300
H	-2.13346300	-3.31086500	-0.26913500
H	-0.91773500	-3.93608200	0.89715400
C	0.18055800	0.61951000	-2.75233900
H	-0.65184400	0.43245200	-3.45603000
H	-0.19687600	1.40789200	-2.07471200
H	1.04158200	1.01750000	-3.31728000
C	3.51496400	-2.96154200	-1.37135600
H	4.29984200	-2.19477800	-1.50425900
H	3.76482500	-3.52730300	-0.45586800
H	3.63083100	-3.67256000	-2.21734300

NCS

Charge: 0 Multiplicity: 1

C	3.77621800	0.27100000	0.05453700
C	3.33843600	-0.99848900	0.43772700
C	2.20139900	-1.15277700	1.23086600
C	1.49051800	-0.01972900	1.65436400

C	1.92753200	1.26258800	1.27609000
C	3.06532600	1.39606100	0.48305700
H	4.65411500	0.38271900	-0.58138600
H	1.85083400	-2.14318200	1.52037500
H	1.36949600	2.14208600	1.59789100
N	0.36599700	-0.17873200	2.43004800
C	-0.56296000	0.27866900	3.03868800
S	-1.80735800	0.78782600	3.85990400
C	4.12990500	-2.21827200	0.02775100
C	3.56643700	2.77337700	0.11628100
F	4.11241700	2.78697400	-1.10985700
F	4.51194100	3.18960400	0.97664900
F	2.57991600	3.68345900	0.13720800
F	5.06789600	-2.51731300	0.94349700
F	3.34306500	-3.29830400	-0.10171900
F	4.76154100	-2.02735700	-1.14089000

INT1

Charge: 0 Multiplicity: 1

C	-0.68261700	2.69967100	-0.30172200
C	-1.80272600	2.53134400	0.63467600
C	-2.84921500	1.72595100	0.30487600
C	-2.91966000	1.04222600	-0.96739800
C	-1.83082000	1.25513800	-1.89438900
C	-0.75491300	2.03726000	-1.61038800

H	-3.63557100	1.54659400	1.03827400
H	-1.89153200	0.75062700	-2.86272200
C	-1.67951800	3.21570000	1.96312600
H	-0.77475300	2.87566200	2.49272400
H	-1.56117200	4.30330300	1.83332600
H	-2.55763700	3.02363000	2.59629500
C	0.38634000	2.22545600	-2.56488200
H	0.72445300	3.27236100	-2.57855800
H	1.24289700	1.60932800	-2.25023800
H	0.10429600	1.92592800	-3.58443500
C	-3.88684000	0.12091400	-1.28757400
H	-3.73714000	-0.44794000	-2.20789700
C	-5.08809100	-0.21419800	-0.52323400
C	-5.48257700	-1.56555400	-0.41933500
C	-5.89073500	0.77235300	0.08510600
C	-6.62222700	-1.91519000	0.30287100
H	-4.83319600	-2.32457800	-0.86007200
C	-7.03791000	0.41665400	0.79552300
H	-5.63111700	1.82528300	-0.03507300
C	-7.40362300	-0.92727200	0.91345700
H	-6.90444400	-2.96710500	0.39154000
H	-7.65485500	1.19452500	1.25166900
H	-8.30048900	-1.20413300	1.47272700
O	0.30299800	3.38343000	0.04167200

C	-0.20000900	-2.08208700	-2.12478700
C	0.55646500	-2.21224100	-0.81115800
C	-1.63771400	-1.82615400	0.20286400
C	-1.58238700	-2.72205800	-1.98222800
H	0.85542900	-3.26794900	-0.64410000
H	1.47189700	-1.60301400	-0.82923900
H	-0.30783000	-1.01177200	-2.36080300
H	0.37493300	-2.54824400	-2.94084800
H	-1.47249200	-3.82640700	-1.96051000
H	-2.18676100	-2.50976100	-2.88416300
C	0.39759000	-1.45641400	1.55243500
C	-0.37750900	-0.40153700	2.32515700
C	-1.80944000	-0.87482600	2.53281600
H	0.10088900	-0.20433400	3.29661300
H	0.50233800	-2.38175500	2.15808900
H	1.40382200	-1.08440600	1.32312100
H	-1.82749100	-1.66352000	3.31337800
H	-2.43730200	-0.04657100	2.90292000
H	-0.36366800	0.52982400	1.74145300
N	-0.26571100	-1.73183600	0.28803800
N	-2.30848100	-2.26792800	-0.81693700
N	-2.35326400	-1.35658200	1.28224300
H	-3.34930500	-1.51506600	1.20315800
C	5.43549500	1.99797200	0.06989400

C	4.38497100	1.06891500	0.04446300
C	4.66694300	-0.29974700	0.11557300
C	5.99144800	-0.73948000	0.21254200
C	7.03863600	0.19518300	0.23818000
C	6.75845100	1.55901300	0.16687200
H	5.20030700	3.06128700	0.01372400
H	3.84659300	-1.01568400	0.09391200
H	8.06317600	-0.17312900	0.31436900
H	7.57372400	2.28552000	0.18703500
C	2.95425900	1.50236300	-0.05709900
C	6.33819600	-2.18569000	0.29038700
O	2.03000500	0.70397000	-0.06135900
O	7.46301300	-2.62064300	0.37512900
O	2.80244300	2.80857800	-0.14932400
H	1.81729400	3.04588200	-0.15833800
O	5.25479300	-2.99044700	0.25723100
H	5.58930200	-3.90105400	0.31271400

TS1

Charge: 0 Multiplicity: 1

C	-0.64559700	2.82406400	0.05646400
C	-1.72389400	2.40141400	0.92769400
C	-2.67682400	1.52568000	0.46409000
C	-2.67421500	1.03823200	-0.87790200
C	-1.68044100	1.56309100	-1.75779500

C	-0.69780600	2.42702900	-1.33956800
H	-3.43871000	1.16668900	1.15797200
H	-1.70031300	1.24422000	-2.80536900
C	-1.68906500	2.89358700	2.34550300
H	-0.76678000	2.56169400	2.85106600
H	-1.66531300	3.99467300	2.37945000
H	-2.55906700	2.53945800	2.91929500
C	0.34574600	2.97339400	-2.27218200
H	0.44732200	4.06321000	-2.14656700
H	1.34112600	2.55050600	-2.06255400
H	0.09585900	2.75769900	-3.32161500
C	-3.43625700	-0.08409300	-1.29139200
H	-3.38300100	-0.31065300	-2.36099400
C	-4.73416300	-0.46526000	-0.66015500
C	-5.16749500	-1.80296100	-0.59296300
C	-5.60894300	0.54070300	-0.20665700
C	-6.41886800	-2.12397300	-0.06603500
H	-4.48842600	-2.58393000	-0.93551100
C	-6.86214400	0.21708400	0.31854500
H	-5.30782900	1.58558000	-0.28701100
C	-7.27087700	-1.11596000	0.39621300
H	-6.73074100	-3.17005100	-0.01509300
H	-7.52320600	1.01518000	0.66471000
H	-8.24972200	-1.36889800	0.81041800

O	0.30163200	3.52793500	0.52199200
C	-0.04333600	-1.50220600	-2.11320200
C	0.63328300	-1.97538400	-0.83852700
C	-1.58788200	-1.65694500	0.17453400
C	-1.46585800	-2.05447700	-2.16983700
H	0.92350500	-3.04060500	-0.91767600
H	1.53870600	-1.38344500	-0.65202700
H	-0.07532300	-0.40340200	-2.10716600
H	0.54321100	-1.82304700	-2.98781400
H	-1.43931800	-3.15306200	-2.30791200
H	-1.99999500	-1.65427900	-3.04769400
C	0.36217900	-1.74804300	1.64178500
C	-0.32863200	-0.67962500	2.47591900
C	-1.80991800	-1.00784300	2.58767200
H	0.12023500	-0.61782700	3.47821700
H	0.30413500	-2.74024000	2.13025300
H	1.41757000	-1.48744600	1.50649700
H	-1.97069900	-1.81085900	3.33050300
H	-2.37289400	-0.12578600	2.93204300
H	-0.17765900	0.28575700	1.97211300
N	-0.24582400	-1.79459100	0.31398400
N	-2.23335100	-1.72954400	-0.98135900
N	-2.32558400	-1.42852200	1.29339300
H	-3.32531600	-1.39825900	1.13569800

C	5.22940700	1.87246600	-0.17765100
C	4.16765500	0.99206000	0.07670900
C	4.42056800	-0.37933700	0.18018900
C	5.72106500	-0.87223200	0.02566400
C	6.77836400	0.01485000	-0.23102500
C	6.52940700	1.38287600	-0.33128000
H	5.01742300	2.93946800	-0.25320900
H	3.59668600	-1.06107400	0.38211800
H	7.78430700	-0.39210000	-0.34749900
H	7.35176900	2.07371100	-0.53014900
C	2.75696600	1.48932300	0.24057900
C	6.02816500	-2.32468700	0.12417000
O	1.82666400	0.71522400	0.43088700
O	7.12973100	-2.80855600	0.00561400
O	2.64757400	2.78868200	0.14237200
H	1.65553600	3.11566700	0.26802600
O	4.93413500	-3.08278700	0.36379800
H	5.24880700	-4.00088400	0.41068200

INT2

Charge: 0 Multiplicity: 1

C	-0.71629400	3.04200700	0.50603000
C	-1.58136300	2.33429700	1.36959500
C	-2.47320400	1.40924300	0.82655500
C	-2.54363700	1.18288400	-0.55497900

C	-1.78002000	2.00432400	-1.39824700
C	-0.87094600	2.93353300	-0.89808800
H	-3.09595300	0.82917400	1.51123800
H	-1.86633300	1.88544700	-2.48359600
C	-1.42493600	2.51852500	2.85296800
H	-0.41027700	2.22255500	3.16699300
H	-1.53389600	3.57597800	3.14118000
H	-2.16267300	1.92473300	3.41490700
C	-0.02097200	3.78192200	-1.80259000
H	-0.07634300	4.84189400	-1.50563500
H	1.04009900	3.49759100	-1.72253500
H	-0.33939100	3.69194600	-2.85209100
C	-3.15009900	-0.06702000	-1.15592800
H	-3.41897800	0.16237300	-2.19770500
C	-4.41536100	-0.61894400	-0.51081900
C	-4.70676400	-1.99184300	-0.52913200
C	-5.36412700	0.26154900	0.03290200
C	-5.90473700	-2.47643200	0.00378800
H	-3.98489000	-2.68554900	-0.96448600
C	-6.56313600	-0.22193800	0.56003400
H	-5.15478600	1.33206400	0.04260400
C	-6.83643400	-1.59288700	0.55284100
H	-6.11044700	-3.54935000	-0.01527900
H	-7.28763400	0.47857700	0.98190100

H	-7.77238800	-1.96987400	0.97137600
O	0.23066000	3.81451700	1.04111700
C	0.00539500	-0.72291300	-2.54316100
C	0.78066300	-1.54356000	-1.52969100
C	-1.33497700	-1.50252500	-0.23999200
C	-1.42439400	-1.24610400	-2.61967000
H	1.16062300	-2.48122900	-1.96998200
H	1.61967600	-0.95115900	-1.14114900
H	-0.01078100	0.32606000	-2.21605400
H	0.50061400	-0.77501400	-3.52395400
H	-1.43650100	-2.30628900	-2.92956800
H	-2.02313700	-0.68635300	-3.34750900
C	0.61137400	-2.43911700	0.80691400
C	0.33487700	-1.51555700	1.98827400
C	-1.16682500	-1.50501300	2.24571700
H	0.86884200	-1.85269800	2.88863000
H	0.24273500	-3.46290100	1.00001000
H	1.68234700	-2.50818000	0.58381700
H	-1.47295500	-2.37159500	2.85550800
H	-1.46133800	-0.58984900	2.78297200
H	0.69523100	-0.51174300	1.71179500
N	-0.06352100	-1.89927300	-0.37985000
N	-2.07559600	-1.11581200	-1.30994200
N	-1.90022400	-1.56079700	0.97789000

H	-2.90150900	-1.40783600	1.01018400
C	4.94673000	1.75721400	-0.33617900
C	3.82218400	1.00184100	0.02073900
C	3.97294600	-0.36644400	0.25260300
C	5.21852200	-0.98933700	0.10929800
C	6.33717800	-0.22594800	-0.26372700
C	6.19602100	1.14425800	-0.48312500
H	4.81145800	2.82894400	-0.49251700
H	3.09908700	-0.93446300	0.55800100
H	7.30155400	-0.72518200	-0.37286900
H	7.06622800	1.74034200	-0.76874400
C	2.44675900	1.64391400	0.16663500
C	5.39336300	-2.44445700	0.33415800
O	1.46718500	0.87242000	0.27816700
O	6.43887300	-3.04744400	0.26162100
O	2.42706600	2.90727400	0.14504300
H	1.14048100	3.54095900	0.63762700
O	4.23144500	-3.08824800	0.63435000
H	4.47808400	-4.01916200	0.76352900

INT3

Charge: -1 Multiplicity: 1

C	1.10501700	-2.93401900	-0.52535900
C	-0.18508200	-2.40090000	-0.45581400
C	-0.48654200	-1.19985700	-1.15508800

C	0.52625400	-0.60651700	-1.95849900
C	1.80132300	-1.17835700	-1.99934400
C	2.12291500	-2.33915600	-1.28492400
H	1.32507700	-3.84963400	0.03563300
H	2.57263400	-0.69245800	-2.60663700
C	-4.09638300	0.53876600	-0.61900100
C	-3.95699700	1.60634100	0.26389900
C	-4.93117300	1.80840200	1.24951800
C	-5.98725600	0.90038400	1.37600200
C	-6.04853900	-0.23385900	0.55887200
C	-5.07866400	-0.43043200	-0.43280000
H	-3.37890000	0.41738900	-1.42241200
H	-4.81720000	2.65312500	1.93181800
H	-6.74318300	1.05186200	2.15131500
H	-6.82388100	-0.99184000	0.68897800
C	-2.69190000	2.41653600	0.25877200
C	-5.01920700	-1.71480600	-1.21568900
O	-1.63110600	1.81488900	-0.28202100
O	-5.99190100	-2.42482900	-1.36427200
O	-2.61480300	3.51745400	0.76058500
H	-1.72054900	0.82071000	-0.57751200
O	-3.82693400	-2.04087900	-1.70730300
H	-3.03276000	-1.48100900	-1.40173300
O	-1.68174100	-0.63928300	-1.05077300

C	3.79709300	0.91766000	0.11240000
C	3.71636700	-0.19330100	0.95009500
C	2.49963800	-0.56438300	1.52335000
C	1.35607000	0.21149900	1.28758300
C	1.42494100	1.33631800	0.44469000
C	2.64382600	1.66918900	-0.13839900
H	4.74564900	1.18997400	-0.35083500
H	2.42339100	-1.44890900	2.15313700
H	0.51678200	1.90648000	0.24170000
N	0.18040600	-0.13192800	1.89799200
C	-0.95014500	-0.09586000	2.27498900
S	-2.42343800	-0.11694500	2.85253100
C	4.96559900	-0.96046900	1.28541400
C	2.74451900	2.82161600	-1.10176500
C	-1.27321600	-3.06657400	0.34239300
H	-1.78499100	-2.35088400	1.00300300
H	-2.05717700	-3.47445300	-0.31802500
H	-0.87482100	-3.89174300	0.95314800
C	0.19197400	0.61829300	-2.76719200
H	-0.69630300	0.43394400	-3.39478800
H	-0.06260000	1.48387900	-2.13628500
H	1.03115300	0.90788300	-3.41604500
C	3.50007800	-2.94879000	-1.36770700
H	4.27694000	-2.17795800	-1.48923100

H	3.74411800	-3.52226700	-0.46043500
H	3.59438400	-3.63948700	-2.22633100
F	3.04268000	2.39370500	-2.34805700
F	3.73700900	3.67039800	-0.75067800
F	1.61682000	3.53459200	-1.18054100
F	5.63149300	-0.39548000	2.31707500
F	4.70817800	-2.23010400	1.64562400
F	5.83084500	-0.99923700	0.25291000

TS2

Charge: -1 Multiplicity: 1

C	0.38409800	-2.50603200	-1.15145700
C	-0.42821100	-1.73855700	-0.31441600
C	-0.38096300	-0.31844800	-0.39778900
C	0.46825500	0.28150400	-1.37004000
C	1.26433900	-0.53341500	-2.18050400
C	1.25090900	-1.92998300	-2.08890200
H	0.34570300	-3.59695500	-1.06097900
H	1.93180200	-0.05370100	-2.90410400
C	-4.05531700	0.49349800	0.00408500
C	-3.89482200	1.36083900	-1.08367700
C	-4.53354600	1.07353700	-2.29575400
C	-5.30279700	-0.08403100	-2.42947600
C	-5.46680200	-0.94026600	-1.34206900
C	-4.84802800	-0.65490100	-0.11443300

H	-3.56030000	0.74135600	0.94240300
H	-4.41545000	1.77927900	-3.12011100
H	-5.78576300	-0.31336400	-3.38282400
H	-6.07963900	-1.84043200	-1.40893900
C	-3.11416400	2.64879900	-0.96908400
C	-5.11782000	-1.60316100	1.02136800
O	-2.14310700	2.68067700	-0.07354300
O	-5.93999200	-2.48684400	0.93463200
O	-3.39734500	3.60680200	-1.65979700
H	-1.76267000	1.75828600	0.15911200
O	-4.42341900	-1.45356200	2.15797800
H	-3.73084500	-0.76119800	2.14274400
O	-1.09521200	0.42091900	0.44529100
C	3.91676600	0.29927500	0.09607500
C	3.46843900	-0.96646900	0.48728800
C	2.35032900	-1.10890700	1.30272700
C	1.63333600	0.02739200	1.71376000
C	2.10635600	1.30448700	1.36288900
C	3.23392100	1.42770100	0.55392400
H	4.77569000	0.40041200	-0.56710400
H	1.98133800	-2.09525700	1.57703700
H	1.55729100	2.18456300	1.69338600
N	0.50008500	-0.10984300	2.48386300
C	-0.67744200	0.23899400	2.41421400

S	-2.06211400	0.58252100	3.18570400
C	4.23444900	-2.18154200	0.04253100
C	3.73001900	2.78556100	0.13733100
C	-1.31898300	-2.39909700	0.70034400
H	-0.84842800	-2.43233100	1.69889200
H	-2.25568600	-1.84592200	0.82631200
H	-1.55549400	-3.43407100	0.41090700
C	0.51579900	1.77671400	-1.52187700
H	-0.33070500	2.15208000	-2.12145600
H	0.44202700	2.28760700	-0.55305400
H	1.44632000	2.09142100	-2.01633300
C	2.10512300	-2.78640500	-2.99005300
H	2.95261500	-2.21544000	-3.39834600
H	2.51842800	-3.65062400	-2.44708300
H	1.52708500	-3.18156500	-3.84536600
F	3.90228500	2.86304900	-1.19961200
F	4.93206100	3.06744300	0.68753800
F	2.89494600	3.77375800	0.49068800
F	5.31345300	-2.40370600	0.82804200
F	3.49602200	-3.30372700	0.08021000
F	4.70490600	-2.04926500	-1.21374700

INT4

Charge: -1 Multiplicity: 1

C	-0.17155300	-2.34925400	1.13180900

C	0.54670100	-1.55880800	0.22731500
C	0.45047000	-0.16333500	0.35927500
C	-0.28276500	0.43592100	1.39708600
C	-0.98758200	-0.40007600	2.26920300
C	-0.96068300	-1.79444100	2.14480100
H	-0.12481600	-3.43689300	1.02390700
H	-1.58950200	0.05857800	3.05941300
C	4.09491600	0.53221800	0.10643900
C	3.49713100	1.23430200	1.15527300
C	3.22972700	0.57240400	2.35929400
C	3.53236800	-0.78437700	2.49697100
C	4.09823100	-1.48722600	1.43317200
C	4.36594600	-0.83212800	0.22252400
H	4.31280700	1.04583600	-0.82617500
H	2.76607800	1.13635200	3.17019500
H	3.30197800	-1.30120000	3.43147100
H	4.32500400	-2.55224500	1.50852400
C	3.08614600	2.67075000	1.00464700
C	4.90195800	-1.62562200	-0.93381900
O	2.91277200	3.12810100	-0.23701300
O	5.53399600	-2.64658700	-0.78125900
O	2.91093700	3.38979700	1.96500600
H	2.82236200	2.42403500	-0.93288500
O	4.63156000	-1.16068800	-2.15950200

H	3.94099000	-0.44568800	-2.19502000
O	1.12546700	0.67727100	-0.50219700
C	-3.81704900	0.10942700	-0.27354100
C	-3.21236600	-1.05526400	-0.75557600
C	-2.03405500	-1.00273500	-1.49547900
C	-1.38092100	0.22607600	-1.72401500
C	-2.02055900	1.40362000	-1.27647300
C	-3.21378100	1.33716900	-0.56044500
H	-4.73255800	0.05956400	0.31507000
H	-1.56324800	-1.91938200	-1.84637500
H	-1.54770100	2.36455800	-1.47515600
N	-0.18516600	0.28888800	-2.40469200
C	0.94236400	0.62528500	-1.88778800
S	2.35525200	1.09884000	-2.75634000
C	-3.85721200	-2.38485700	-0.48063800
C	-3.84408600	2.58721500	-0.01242300
C	1.33807500	-2.20031400	-0.87896700
H	0.71399100	-2.36228400	-1.77406000
H	2.17265200	-1.57262600	-1.20323900
H	1.73795500	-3.17418500	-0.56120200
C	-0.30734600	1.93122100	1.55945900
H	0.51015600	2.27519000	2.21425500
H	-0.16070700	2.44085800	0.59911600
H	-1.25968600	2.26227300	1.99617500

C	-1.79955400	-2.66953400	3.04195700
H	-1.92116000	-2.22648000	4.04289600
H	-2.80848800	-2.80625100	2.61691600
H	-1.35539000	-3.67003300	3.16121300
F	-3.71385300	2.66397300	1.33306300
F	-5.17114000	2.63098200	-0.25936800
F	-3.30860300	3.70804400	-0.52050200
F	-4.79973100	-2.69454400	-1.39763800
F	-2.97275600	-3.39822300	-0.48134400
F	-4.47986700	-2.40496600	0.72007700

INT5

Charge: -1 Multiplicity: 1

C	-2.81593600	2.75967400	-2.19824800
C	-2.31426200	1.46145800	-2.03220800
C	-1.82480900	1.10262800	-0.76982700
C	-1.81586000	1.99985800	0.31138600
C	-2.32995400	3.28228700	0.09539000
C	-2.83470700	3.68388400	-1.14889500
H	-3.20257100	3.05143200	-3.17962000
H	-2.34043800	3.98816900	0.93211800
C	-3.45325500	-1.86889400	0.88373900
C	-3.64889900	-1.06088900	2.00473300
C	-4.54585900	0.01460700	1.93112500
C	-5.20655000	0.29252700	0.73452400

C	-4.97677000	-0.49896800	-0.39310900
C	-4.08577400	-1.57636500	-0.32646100
H	-2.76603600	-2.70961600	0.93927800
H	-4.69189400	0.63082600	2.82007500
H	-5.88504400	1.14635900	0.67136000
H	-5.46209000	-0.28517400	-1.34675400
C	-2.88808000	-1.28594000	3.27906000
C	-3.78576900	-2.34910600	-1.57810100
O	-1.69015300	-1.87542900	3.16927300
O	-4.55566300	-2.37490300	-2.51478400
O	-3.31197200	-0.93900500	4.35771500
H	-1.34628100	-1.95489900	2.24316400
O	-2.62221700	-2.99947000	-1.62780200
H	-1.95390400	-2.71842500	-0.94704000
O	-1.47444500	-0.22083800	-0.58171500
C	4.89692100	-0.00802300	0.06965500
C	4.20028100	1.17217400	0.34262800
C	2.81791400	1.24074700	0.17168100
C	2.07764800	0.12018900	-0.26179400
C	2.79035100	-1.06818500	-0.54486500
C	4.17487700	-1.11643500	-0.38458200
H	5.97506300	-0.06553600	0.21426600
H	2.27469700	2.16415800	0.37443900
H	2.24265600	-1.95014800	-0.87105500

N	0.72294400	0.29195400	-0.43635600
C	-0.18810100	-0.60265500	-0.27639500
S	-0.12143800	-2.23923100	0.29436300
C	4.95860900	2.40024500	0.76306200
C	4.92586900	-2.36672200	-0.75573200
C	-2.31986600	0.46650400	-3.16201300
H	-2.36276300	0.98101500	-4.13372500
H	-1.42474500	-0.17063600	-3.13868500
H	-3.18931900	-0.20849400	-3.10567600
C	-1.26673500	1.60386500	1.65416000
H	-0.18445100	1.80506100	1.70069800
H	-1.76135100	2.15861700	2.46492600
H	-1.38994500	0.53196500	1.84365600
C	-3.36048900	5.08449000	-1.34811300
H	-3.93243300	5.17123500	-2.28451100
H	-4.01906900	5.39053200	-0.51871400
H	-2.53837100	5.82052600	-1.39337600
F	5.99164200	-2.57218500	0.04785000
F	5.41235900	-2.30051200	-2.01625900
F	4.16243200	-3.46825600	-0.69431600
F	4.23248300	3.20339800	1.56273700
F	6.09192000	2.09588500	1.42800000
F	5.33044500	3.15127700	-0.29761400

INT6

Charge: -1 Multiplicity: 1

C	-4.58372400	0.00136000	1.33174500
C	-4.99961900	1.15502400	0.53484300
C	-4.09691000	2.13811100	0.24252300
C	-2.72659200	2.07621500	0.67643900
C	-2.31574500	0.90670500	1.40981500
C	-3.19195900	-0.07868400	1.76762200
H	-4.40860100	2.97501900	-0.38507600
H	-1.26140300	0.82597200	1.68023900
C	-6.42188000	1.18672800	0.05379200
H	-6.64174900	0.34276500	-0.61996900
H	-7.12227000	1.08171500	0.89869800
H	-6.64158000	2.12337800	-0.47907800
C	-2.79352100	-1.28873800	2.56012000
H	-3.36041100	-1.34153200	3.50452500
H	-3.03294700	-2.21037400	2.00837100
H	-1.71847800	-1.28114900	2.78602700
C	-1.78082200	3.04773600	0.38086100
H	-0.74672500	2.80621900	0.62611500
C	-2.03992000	4.43439600	-0.02382000
C	-1.09919300	5.14081700	-0.80390600
C	-3.17075900	5.13661100	0.44432800
C	-1.29764500	6.48125600	-1.12385400
H	-0.24203000	4.59173800	-1.19648200

C	-3.36678600	6.48071900	0.12174400
H	-3.87979300	4.63103900	1.10066900
C	-2.43511100	7.15840100	-0.66791100
H	-0.56199100	7.00190600	-1.74228200
H	-4.24885600	7.00328100	0.50097300
H	-2.58990100	8.21019700	-0.92231700
O	-5.38602800	-0.92575400	1.60625100
C	-2.78085100	-2.48702200	-0.78149600
C	-4.17191500	-2.33372200	-0.79490500
C	-5.00275800	-3.40700600	-0.45287600
C	-4.43049000	-4.64818400	-0.14239200
C	-3.04266000	-4.79985600	-0.11374600
C	-2.21618400	-3.71909100	-0.42101100
H	-4.59148200	-1.36834800	-1.07262500
H	-5.09507600	-5.48423600	0.08421500
H	-2.60343600	-5.76449200	0.15216000
H	-1.12854100	-3.80530800	-0.39178500
C	-6.50576800	-3.28980400	-0.44687200
C	-1.85591100	-1.35269800	-1.08937200
O	-7.21061000	-4.19361400	-0.83865400
O	-0.66030800	-1.41898200	-0.86333900
O	-7.01294100	-2.14482800	0.00232800
H	-6.37608600	-1.62968600	0.59078400
O	-2.45199800	-0.29563900	-1.61285700

H	-1.77502000	0.43562000	-1.75709700
C	4.21350600	2.20708200	1.41993500
C	3.00539800	2.32459700	0.72394600
C	2.06727500	1.28744100	0.85384000
C	2.27706200	0.19910600	1.71465200
C	3.50387100	0.12535900	2.38401700
C	4.49366800	1.10411500	2.23457000
H	4.96634300	2.99162100	1.30184700
H	3.69612600	-0.74078300	3.02417500
O	0.88669900	1.34940200	0.13229100
C	4.77074400	-2.03845700	-0.42844800
C	5.12888700	-0.74738800	-0.82541500
C	4.18110800	0.14286600	-1.32790400
C	2.82128800	-0.22064800	-1.39780900
C	2.46617200	-1.54002200	-1.03496300
C	3.43048900	-2.42107900	-0.55424400
H	5.51545000	-2.72633900	-0.03021900
H	4.47130100	1.15161500	-1.61837200
H	1.41997500	-1.83153000	-1.10437500
N	1.87358600	0.64949200	-1.88616300
C	0.92695600	1.24242100	-1.26744100
S	-0.40423700	2.03378800	-2.04118800
C	6.56597600	-0.31804100	-0.73965400
C	3.02076100	-3.78438700	-0.07074300

C	2.72986000	3.50245900	-0.17115500
H	2.74182200	3.21289500	-1.23486300
H	1.73240600	3.92583400	0.02118800
H	3.48075300	4.29152200	-0.02050100
C	1.21917500	-0.85216100	1.91168000
H	0.58247700	-0.59197300	2.77582700
H	0.56290200	-0.94586700	1.03795000
H	1.67405800	-1.83028600	2.11789800
C	5.83693600	0.94704800	2.90122300
H	5.73558500	0.58561900	3.93721600
H	6.45841700	0.21558000	2.35859100
H	6.39080200	1.89773000	2.92204400
F	2.91195000	-3.82477600	1.28070000
F	3.92661600	-4.73080600	-0.39664800
F	1.83642500	-4.18152600	-0.56429400
F	7.24556000	-0.56882200	-1.87939800
F	6.69635400	1.00159100	-0.50082400
F	7.23172800	-0.96217100	0.24634900

TS3

Charge: -1 Multiplicity: 1

C	-4.64824200	0.13795100	1.13811200
C	-4.90562300	1.21582800	0.20419400
C	-3.91817300	2.13515400	-0.07577000
C	-2.63117400	2.07811900	0.53010100

C	-2.37407800	0.99477400	1.41739300
C	-3.33232900	0.05798700	1.73674400
H	-4.12204400	2.91671200	-0.81249400
H	-1.37261800	0.90617200	1.84589600
C	-6.26316300	1.26941000	-0.43871100
H	-6.46162500	0.37608500	-1.05393900
H	-7.05765500	1.27969900	0.32626600
H	-6.37064400	2.15958100	-1.07674900
C	-3.07027600	-1.09011400	2.66797700
H	-3.77644800	-1.08057600	3.51493100
H	-3.22941200	-2.05146200	2.15329600
H	-2.04124700	-1.06546100	3.05682600
C	-1.57542900	2.98714600	0.21728300
H	-0.65694900	2.81239000	0.77939100
C	-1.80077900	4.42677300	-0.10727400
C	-0.80911000	5.20478300	-0.73724700
C	-2.96324500	5.08515700	0.33778900
C	-0.97878700	6.57447900	-0.92923000
H	0.08830400	4.70738200	-1.10624200
C	-3.13600100	6.45781300	0.13993400
H	-3.72691900	4.51658100	0.86774300
C	-2.14784500	7.21007800	-0.49685400
H	-0.19418500	7.14963900	-1.42789900
H	-4.05030300	6.94025800	0.49542500

H	-2.28405500	8.28333500	-0.65340200
O	-5.52862300	-0.73790500	1.41258100
C	-2.81705600	-2.45487500	-0.68272100
C	-4.20602900	-2.30384800	-0.76245300
C	-5.05300200	-3.34559600	-0.37179800
C	-4.49603600	-4.55946500	0.05170900
C	-3.10999800	-4.71069200	0.14231300
C	-2.26791900	-3.65661700	-0.21156900
H	-4.61268500	-1.36130300	-1.12166600
H	-5.17256700	-5.37443300	0.31677600
H	-2.68481400	-5.65372000	0.49488000
H	-1.18246900	-3.74403300	-0.13750100
C	-6.55680600	-3.21553500	-0.42836900
C	-1.89260200	-1.33934700	-1.02918000
O	-7.24993200	-4.15923800	-0.74967800
O	-0.70746300	-1.35281700	-0.74157900
O	-7.05904800	-2.03059300	-0.12476900
H	-6.43158800	-1.45116500	0.45153300
O	-2.47228200	-0.33784700	-1.67602400
H	-1.82372700	0.40907700	-1.76357900
C	4.30586800	1.96623000	1.56661500
C	3.08114300	2.18002600	0.92531400
C	2.10614400	1.17670000	1.02931200
C	2.28709100	0.02800200	1.81150300

C	3.53442200	-0.14218000	2.42441600
C	4.56215700	0.80009300	2.29766500
H	5.08934200	2.72285700	1.46992400
H	3.70921100	-1.05507500	3.00092500
O	0.90128500	1.33759900	0.35371500
C	4.69925500	-2.05732100	-0.58804200
C	5.04730700	-0.75388200	-0.94851300
C	4.07928500	0.16357000	-1.35725200
C	2.71902700	-0.19303900	-1.36849700
C	2.37000000	-1.52157400	-1.04642900
C	3.35167400	-2.42706400	-0.65352400
H	5.45854100	-2.76549300	-0.25876500
H	4.36096800	1.18308400	-1.61726300
H	1.31856300	-1.80219000	-1.06916900
N	1.74675300	0.71371700	-1.75511100
C	0.88125900	1.29174900	-1.02443000
S	-0.46957100	2.16384900	-1.72137900
C	6.49013300	-0.33404800	-0.92262400
C	2.95535600	-3.80173600	-0.18911000
C	2.82513300	3.42280400	0.11574800
H	2.77843300	3.20116200	-0.96352100
H	1.86112500	3.88317600	0.38109700
H	3.62061500	4.16414300	0.27724000
C	1.18332400	-0.97843700	1.98747300

H	0.55500800	-0.70524500	2.85345100
H	0.52008100	-1.02749300	1.11520800
H	1.59587100	-1.97874700	2.17553400
C	5.92230900	0.54105700	2.89413100
H	5.84795100	0.02133500	3.86221900
H	6.52487900	-0.09554500	2.22482800
H	6.48136200	1.47659400	3.04762300
F	2.91393900	-3.87491200	1.16387000
F	3.83751100	-4.74251600	-0.58574900
F	1.74566900	-4.17465200	-0.63448000
F	7.09569200	-0.50980200	-2.11565800
F	6.63929000	0.96721700	-0.60622100
F	7.20933800	-1.04204200	-0.02306600

INT7

Charge: -1 Multiplicity: 1

C	-4.69148200	0.25479000	0.96622700
C	-4.83419700	1.26692100	-0.04385700
C	-3.77552600	2.12202500	-0.33026500
C	-2.54740400	2.04609400	0.35416700
C	-2.40706600	1.05480700	1.34147600
C	-3.43553300	0.17293700	1.65500200
H	-3.90500400	2.87180300	-1.11832800
H	-1.44909100	0.95721500	1.86083900

C	-6.14901200	1.35008200	-0.76915900
H	-6.36148800	0.43262300	-1.34396300
H	-6.98375700	1.44217800	-0.05356400
H	-6.17641900	2.20578100	-1.46169100
C	-3.28022000	-0.90903000	2.68495100
H	-4.05770900	-0.82948300	3.46318800
H	-3.41606400	-1.90353000	2.22802700
H	-2.28790500	-0.87493100	3.16196200
C	-1.37500200	2.90197200	-0.00051800
H	-0.60264500	2.77618100	0.76685200
C	-1.61446400	4.39714100	-0.17324200
C	-0.71123100	5.22494400	-0.86054100
C	-2.70665200	5.00269000	0.46995700
C	-0.89443800	6.60863100	-0.90960600
H	0.14065300	4.77453100	-1.37250400
C	-2.89114800	6.38665700	0.42063400
H	-3.41380200	4.37597500	1.01345200
C	-1.98828300	7.19788200	-0.27071300
H	-0.17751800	7.22808500	-1.45518000
H	-3.75176600	6.83151700	0.92715500
H	-2.13656800	8.28007000	-0.31307600
O	-5.63777500	-0.57334300	1.25276500
C	-2.84856200	-2.44539200	-0.59208000
C	-4.23235000	-2.28381300	-0.72692900

C	-5.10733300	-3.28147400	-0.29072900
C	-4.58103400	-4.46918000	0.23304400
C	-3.19993400	-4.63268000	0.37697000
C	-2.33015100	-3.61734200	-0.02074200
H	-4.61583800	-1.36231800	-1.15696800
H	-5.27884400	-5.25332800	0.53389100
H	-2.80065100	-5.55466400	0.80742200
H	-1.24896700	-3.71545200	0.09336600
C	-6.60882400	-3.12034000	-0.40384700
C	-1.91399400	-1.35964300	-0.98025900
O	-7.30791400	-4.08558600	-0.65418300
O	-0.74312600	-1.32049900	-0.63576100
O	-7.07713100	-1.90545000	-0.23599500
H	-6.44378400	-1.27216800	0.34854000
O	-2.46540800	-0.42654200	-1.75068900
H	-1.84897900	0.33633500	-1.80785800
C	4.36038900	1.85102000	1.61768700
C	3.12291800	2.09828500	1.01366200
C	2.14104300	1.10430500	1.12272900
C	2.31804400	-0.06624500	1.87007700
C	3.57957700	-0.26869900	2.44353200
C	4.61720800	0.66251800	2.31193700
H	5.15192200	2.59884900	1.52018200
H	3.75577600	-1.19725300	2.99358500

O	0.91746400	1.29720400	0.47774600
C	4.64391800	-2.07579600	-0.66317200
C	4.99063100	-0.77010400	-1.01366300
C	4.01549200	0.15872100	-1.37982300
C	2.65745800	-0.19488000	-1.35647900
C	2.30585300	-1.52278500	-1.04504500
C	3.29363900	-2.43885500	-0.69331100
H	5.40823100	-2.79250700	-0.36515800
H	4.29466100	1.18118500	-1.63106400
H	1.25147400	-1.79526000	-1.03787600
N	1.67334400	0.73315100	-1.68558400
C	0.86961800	1.29014100	-0.87927100
S	-0.48437300	2.21344400	-1.55169300
C	6.43587800	-0.35583100	-1.02038100
C	2.90510400	-3.81762100	-0.23155800
C	2.86401500	3.36223900	0.23764900
H	2.83613900	3.17251200	-0.84872000
H	1.89323600	3.80887400	0.50200000
H	3.65092800	4.10512000	0.42981800
C	1.19967300	-1.05493500	2.05245100
H	0.57465900	-0.76524200	2.91517900
H	0.53136900	-1.10002300	1.18332100
H	1.59832200	-2.05956800	2.24697200
C	5.98870300	0.37033600	2.86534900

H	5.93305100	-0.20123200	3.80471400
H	6.57577800	-0.22921200	2.14952600
H	6.55282200	1.29556400	3.05759500
F	2.92758300	-3.90708200	1.12015900
F	3.76025900	-4.75787700	-0.68294800
F	1.67370800	-4.17064600	-0.62590300
F	7.00049000	-0.49414000	-2.23714000
F	6.59734600	0.93442700	-0.66707300
F	7.17878300	-1.09408300	-0.16713800

INT8

Charge: -1 Multiplicity: 1

C	1.43218300	-3.33320500	1.51550900
C	1.19753400	-3.48777200	0.12897200
C	1.90763400	-2.68822100	-0.76841400
C	2.85942200	-1.75653900	-0.33726400
C	3.12461800	-1.66381600	1.03606100
C	2.42953800	-2.43790100	1.96511500
H	1.69232600	-2.77104700	-1.83822700
H	3.84114400	-0.92022900	1.39619300
C	0.16549400	-4.47887800	-0.33602200
H	0.38818100	-5.48405000	0.06110600
H	-0.83565900	-4.21856600	0.03718800
H	0.13098900	-4.53464200	-1.43497900
C	2.61927000	-2.26612000	3.44601500

H	3.39322000	-1.51517400	3.66834900
H	1.66986600	-1.94973700	3.90882200
H	2.89397900	-3.21615300	3.93322000
C	3.51518800	-0.84674500	-1.33929700
H	2.94173300	-0.88154100	-2.27545300
C	4.98357100	-1.09089400	-1.65231100
C	5.58692700	-0.40101100	-2.71815500
C	5.75504200	-2.00728700	-0.92666200
C	6.92468200	-0.61753400	-3.04715700
H	4.99438400	0.32020200	-3.28706500
C	7.09718800	-2.22517000	-1.25499800
H	5.29232700	-2.55700000	-0.10642300
C	7.68801000	-1.53229700	-2.31314400
H	7.37446000	-0.07177100	-3.88093500
H	7.68218700	-2.94587400	-0.67752200
H	8.73697500	-1.70418900	-2.56847000
O	0.72383800	-4.01099500	2.42510800
C	-4.78308700	0.17406700	1.33958700
C	-3.47976200	-0.33088500	1.44248000
C	-3.26140400	-1.67968000	1.73229200
C	-4.36125700	-2.52362300	1.92585600
C	-5.66505700	-2.02822800	1.82022000
C	-5.88020600	-0.68310000	1.52479600
H	-2.61282400	0.30779800	1.28333200

H	-4.15744100	-3.57277700	2.14733800
H	-6.51840600	-2.69719800	1.96178900
H	-6.88677300	-0.27170000	1.42896500
C	-1.83263700	-2.21159900	1.80054300
C	-5.04545900	1.59770200	1.01428200
O	-0.91875300	-1.42020800	1.51806800
O	-6.14176500	2.09108700	0.85435900
O	-1.72048800	-3.43754300	2.12010800
H	-0.29357700	-3.84084700	2.28097300
O	-3.91424800	2.32910500	0.90235000
H	-4.17807800	3.22079700	0.62398900
C	2.31292700	2.71782700	3.18346600
C	1.66115700	2.16451200	2.07608700
C	1.84314200	2.81238500	0.84433600
C	2.60797000	3.97194200	0.69136400
C	3.23372400	4.48870400	1.83422100
C	3.10417800	3.87174000	3.08368400
H	2.19073000	2.22994700	4.15497700
H	3.84146900	5.39335100	1.73899000
O	1.18262400	2.30593200	-0.27651000
C	-3.30862000	0.42426700	-1.92736800
C	-2.59932200	1.62216900	-1.95500900
C	-1.21124400	1.64143200	-1.78491300
C	-0.50823900	0.44204100	-1.58742000

C	-1.22832200	-0.76844200	-1.54424500
C	-2.60816600	-0.76927900	-1.72101000
H	-4.39141100	0.41621300	-2.04035900
H	-0.67138000	2.58648500	-1.80072800
H	-0.68201600	-1.68888800	-1.34816900
N	0.87961800	0.36296900	-1.49849300
C	1.63398700	1.17005200	-0.88459300
S	3.39410200	0.94394300	-0.79382200
C	-3.31354600	2.92040400	-2.20048000
C	-3.36277100	-2.07101000	-1.77637300
C	0.79506100	0.94367700	2.17140800
H	0.65387100	0.62959600	3.21372400
H	-0.19537700	1.10986800	1.72573900
H	1.21251900	0.08196700	1.62968800
C	2.75283700	4.61518500	-0.66231600
H	1.77044100	4.87284200	-1.08970600
H	3.35650200	5.53215900	-0.60026400
H	3.23994500	3.92942800	-1.37470900
C	3.79775400	4.42619900	4.30372700
H	3.08244700	4.61468700	5.12152800
H	4.54854500	3.71722000	4.69230900
H	4.31397900	5.37207200	4.08100200
F	-2.77184300	-3.04491300	-1.07264900
F	-3.45788300	-2.51898000	-3.05265600

F	-4.62436300	-1.94421000	-1.32692000
F	-3.04821000	3.41766900	-3.42757200
F	-4.64937600	2.80333700	-2.10636000
F	-2.94294000	3.88414500	-1.32281000

References

1. Uno, T.; Minari, M.; Kubo, M.; Itoh, T. Asymmetric Anionic Polymerization of 2,6-Dimethyl-7phenyl-1,4-benzoquinone Methide. *J. Polym. Sci. Part A: Polym. Chem.* **2004**, *42*, 4548.
2. Gaussian 16, Revision C.02, M. J. Frisch, G. W. Trucks, H. B. Schlegel, G. E. Scuseria, M. A. Robb, J. R. Cheeseman, G. Scalmani, V. Barone, G. A. Petersson, H. Nakatsuji, X. Li, M. Caricato, A. V. Marenich, J. Bloino, B. G. Janesko, R. Gomperts, B. Mennucci, H. P. Hratchian, J. V. Ortiz, A. F. Izmaylov, J. L. Sonnenberg, D. Williams-Young, F. Ding, F. Lipparini, F. Egidi, J. Goings, B. Peng, A. Petrone, T. Henderson, D. Ranasinghe, V. G. Zakrzewski, J. Gao, N. Rega, G. Zheng, W. Liang, M. Hada, M. Ehara, K. Toyota, R. Fukuda, J. Hasegawa, M. Ishida, T. Nakajima, Y. Honda, O. Kitao, H. Nakai, T. Vreven, K. Throssell, J. A. Montgomery, Jr., J. E. Peralta, F. Ogliaro, M. J. Bearpark, J. J. Heyd, E. N. Brothers, K. N. Kudin, V. N. Staroverov, T. A. Keith, R. Kobayashi, J. Normand, K. Raghavachari, A. P. Rendell, J. C. Burant, S. S. Iyengar, J. Tomasi, M. Cossi, J. M. Millam, M. Klene, C. Adamo, R. Cammi, J. W. Ochterski, R. L. Martin, K. Morokuma, O. Farkas, J. B. Foresman, and D. J. Fox, Gaussian, Inc., Wallingford CT, 2019.
3. (a) Neese, F.; Wennmohs, F.; Becker, U.; Riplinger, C. The ORCA quantum chemistry program package. *J. Chem. Phys.* **2020**, *152*, 224108; (b) Neese, F. Software update: The ORCA program system—Version 5.0. *Computational Molecular Science.* **2022**, *12*, e1606; (c) Neese, F.; Wennmohs, F.; Hansen, A.; Becker, U.; Efficient, approximate and parallel Hartree–Fock and hybrid DFT calculations. A 'chain-of-spheres' algorithm for the Hartree–Fock exchange. *Chem. Phys.* **2009**, *356*, 98; (d) Izsák, R.; Neese, F. An overlap fitted chain of spheres exchange method. *J. Chem. Phys.* **2011**, *135*, 144105; (e) Izsák, R.; Neese, F.; Klopper, W.; Robust fitting techniques in the chain of spheres approximation to the

Fock exchange: The role of the complementary space. *J. Chem. Phys.* **2013**, *139*, 094111; (f) Helmich-Paris, B.; de Souza, B.; Neese, F.; Izsák, R. An improved chain of spheres for exchange algorithm. *J. Chem. Phys.* **2021**, *155*, 104109. (g) Hattig, C. Optimization of auxiliary basis sets for RI-MP2 and RI-CC2 calculations: Core–valence and quintuple-f basis sets for H to Ar and QZVPP basis sets for Li to Kr. *Phys. Chem. Chem. Phys.* **2005**, *7*, 59.

4. Lee, C.; Yang, W.; Parr, R. G. Development of the Colle-Salvetti correlation-energy formula into a functional of the electron density. *Phys Rev B Condens Matter*. **1988**, *37*, 785.
5. Weigend, F.; Ahlrichs, R. Balanced basis sets of split valence, triple zeta valence and quadruple zeta valence quality for H to Rn: Design and assessment of accuracy. *Phys Chem Chem Phys.* **2005**, *7*, 3297.
6. Grimme, S.; Antony, J.; Ehrlich, S.; Krieg, H. A consistent and accurate *ab initio* parametrization of density functional dispersion correction (DFT-D) for the 94 elements H-Pu. *J Chem Phys.* **2010**, *132*, 154104.
7. (a) Gonzalez, C.; Schlegel, H. B. Reaction path following in mass-weighted internal coordinates. *J. Phys. Chem.* **1990**, *94*, 5523; (b) Fukui, K. The path of chemical reactions - the IRC approach. *Acc. Chem. Res.* **1981**, *14*, 363.
8. Grimme, S. Semiempirical hybrid density functional with perturbative second-order correlation. *J. Chem. Phys.* **2006**, *124*, 034108.
9. (a) Rappoport, D.; Furche, F. Property-optimized Gaussian basis sets for molecular response calculations. *J. Chem. Phys.* **2010**, *133*, 134105; (b) Rappoport, D. Property-optimized Gaussian basis sets for lanthanides. *J. Chem. Phys.* **2021**, *155*, 124102.
10. Caldeweyher, E.; Bannwarth, C.; Grimme, S. Extension of the D3 dispersion coefficient model. *J. Chem. Phys.* **2017**, *147*, 034112.
11. Marenich, A. V.; Cramer, C. J.; Truhlar, D. G. Universal Solvation Model Based on Solute Electron Density and on a Continuum Model of the Solv. *J. Phys. Chem.* **2009**, *113*, 6378.
